# Mitochondrial Capture Misleads about Ecological Speciation in the *Daphnia* *pulex* Complex

**DOI:** 10.1371/journal.pone.0069497

**Published:** 2013-07-15

**Authors:** Silvia Marková, France Dufresne, Marina Manca, Petr Kotlík

**Affiliations:** 1 Institute of Animal Physiology and Genetics, Laboratory of Molecular Ecology, Academy of Sciences of the Czech Republic, Liběchov, Czech Republic; 2 Département de Biologie, Centre d’Études Nordiques, Université du Québec à Rimouski, Québec, Canada; 3 CNR Istituto per lo Studio degli Ecosistemi, Verbania, Italy; Consiglio Nazionale delle Ricerche (CNR), Italy

## Abstract

The North American ecological species *Daphnia* *pulicaria* and *Daphnia* *pulex* are thought to have diverged from a common ancestor by adaptation to sympatric but ecologically distinct lake and pond habitats respectively. Based on mtDNA relationships, European *D* *. pulicaria* is considered a different species only distantly related to its North American counterpart, but both species share a lactate dehydrogenase (*Ldh*) allele F supposedly involved in lake adaptation in North America, and the same allele is also carried by the related Holarctic *Daphnia* *tenebrosa* . The correct inference of the species’ ancestral relationships is therefore critical for understanding the origin of their adaptive divergence. Our species tree inferred from unlinked nuclear loci for *D* *. pulicaria* and *D* *. pulex* resolved the European and North American *D* *. pulicaria* as sister clades, and we argue that the discordant mtDNA gene tree is best explained by capture of *D* *. pulex* mtDNA by *D* *. pulicaria* in North America. The *Ldh* gene tree shows that F-class alleles in *D* *. pulicaria* and *D* *. tenebrosa* are due to common descent (as opposed to introgression), with *D* *. tenebrosa* alleles paraphyletic with respect to *D* *. pulicaria* alleles. That *D* *. tenebrosa* still segregates the ancestral and derived amino acids at the two sites distinguishing the pond and lake alleles suggests that *D* *. pulicaria* inherited the derived states from the *D* *. tenebrosa* ancestry. Our results suggest that some adaptations restricting the gene flow between *D* *. pulicaria* and *D* *. pulex* might have evolved in response to selection in ancestral environments rather than in the species’ current sympatric habitats. The Arctic ( *D* *. tenebrosa* ) populations are likely to provide important clues about these issues.

## Introduction

The concept of ecological speciation posits that reproductive barriers between species can evolve as a direct result of ecologically-based divergent selection, as opposed to accumulation of genetic incompatibilities in geographical isolation as an indirect by-product of random processes such as genetic drift [1]. However, ecological speciation can occur under various geographical settings, including in allopatry, and it can be either entirely allopatric, including the evolution of reproductive isolation by adaptation to different environments, or partially allopatric, with reproductive isolation evolving upon secondary contact by reduced hybrid fitness (reinforcement) or fitness costs experienced during heterospecific encounters [2,3].

Although the model of ecological speciation has gained theoretical [3,4] and empirical support [2,5], it remains unclear how often the divergence between sympatric ecological species was initiated by selection between geographically non-overlapping environments and the species’s sympatric occurrence is due to secondary contact rather than to divergence in sympatry [1,6]. Distinguishing between these alternatives is important to identify the source of ecological selection that triggered the species divergence and the mechanisms by which the reproductive isolation evolved (e.g., [7,8]). Inferring the geography of speciation can be however challenging [2,6,9], and cases where the speciation is on-going therefore would hold the promise that the geographic context of the adaptive divergence can be more directly observed [1].

**Figure 1:**
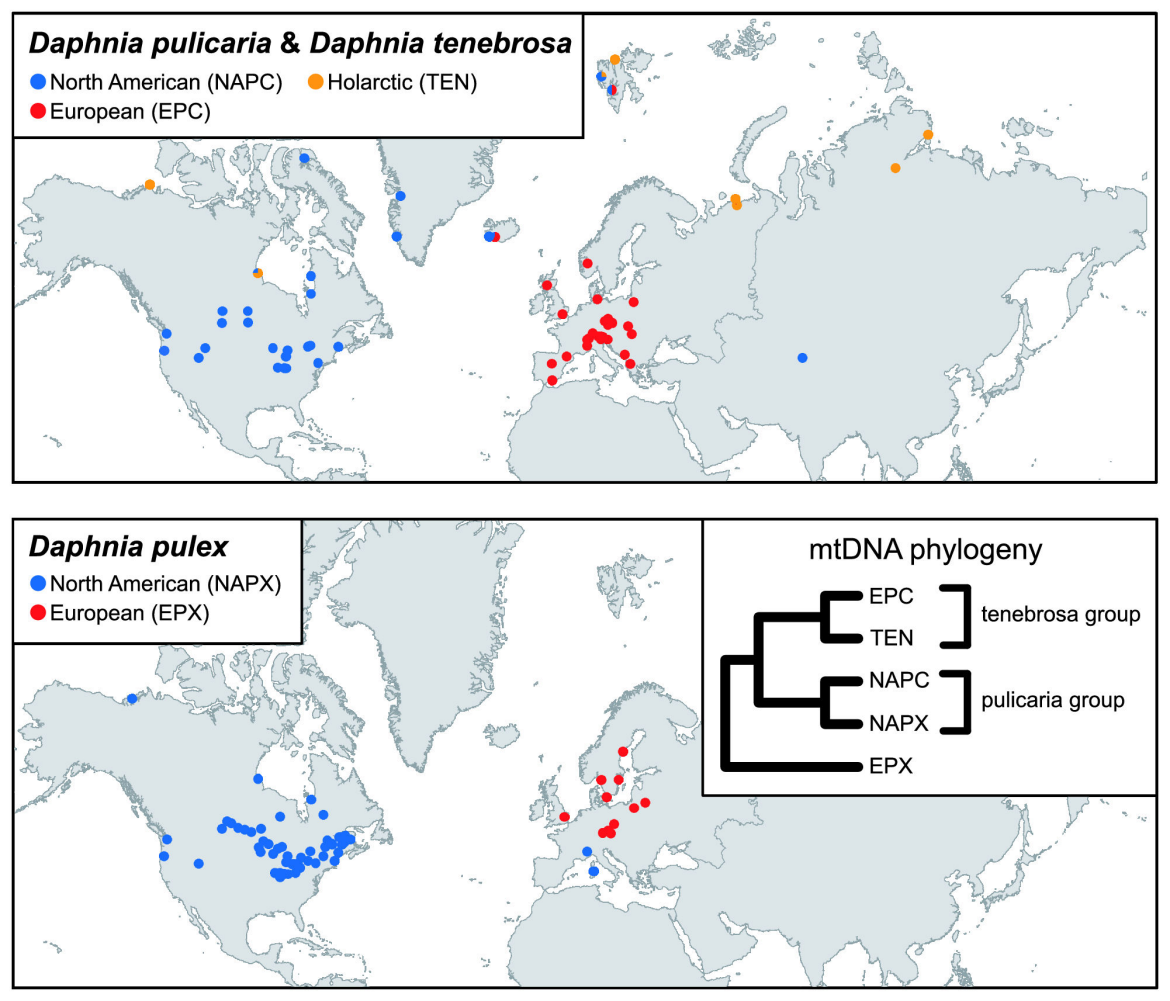
Geographic distribution of the collection sites for *Daphnia pulicaria*, *Daphnia pulex* and *Daphnia tenebrosa*. Inset: A phylogenetic hypothesis for the species based on mitochondrial DNA (mtDNA).

*Daphnia* *pulex* and *Daphnia* *pulicaria* are ecologically distinct cladoceran crustaceans thought to be in the process of ecological speciation [10,11]. They are sympatric over a large part of North America and Europe (Figure 1) and the main argument supporting the ecologically-based divergent selection as the driving force of their divergence is that they have undergone genetic differentiation even though they often coexist in adjacent but ecologically divergent habitats: *D* *. pulex* in shallow, fishless temporary ponds and *D* *. pulicaria* in deep lakes and reservoirs [11–13]. It has been suggested that divergent selective pressures between the pond and lake habitats resulted in marked differences in life history traits between the two species, with *D* *. pulex* tolerating higher temperatures, growing faster, and reaching sexual maturity at an earlier age than *D* *. pulicaria* , which have longer life span, slower metabolic rate and are more efficient grazers able to thrive in low-nutrient lakes [12,14–16]. These life-history differences are thought to restrict the gene flow between the ponds and lakes, as suggested by a strong genetic cohesion within each habitat and significant genetic differentiation between them [11]. However, the two species readily hybridize in nature, thus leading to the suggestion that they may still be in the process of diverging from the common ancestor, with the differences between the pond and lake habitats being the source of the divergent selection [10,17]. This view is supported by inferred levels of gene flow considered high enough to prevent divergence in an absence of strong local selection [10,11]. Interestingly, the introgression appears to be asymmetrical, with heterozygotes for pond and lake alleles found in ponds but rarely in lakes, while many lake *Daphnia* carry mtDNA haplotypes characteristic of pond populations, but not vice versa [11]. Therefore, there appears to be occasional dispersal of *D* *. pulicaria* into ponds followed by asymmetric hybridization involving males produced by the dispersers and females of the residents, explaining the absence of *D* *. pulicaria* mtDNA in ponds [11]. *Daphnia* *pulex* are maladapted to lake habitats [11], but if some backcrosses survive in lakes due to the presence of lake-adapted alleles in their genome, it might explain the introgression of *D* *. pulex* mtDNA in lake populations [11].

Essentially all of the insights into the ecological divergence and speciation of *D* *. pulex* and *D* *. pulicaria* have been obtained in North America and it is unknown to what extent the same scenario applies to the European populations (e.g., [10]). The current view however is that the names *D* *. pulex* and *D* *. pulicaria* are used for different species in North America than in Europe (Figure 1). The reasons are historical as both species were assumed to have a broad Holarctic distribution [18], and only with the accumulation of genetic data has it become clear that the North American *D* *. pulicaria* and *D* *. pulex* are genetically distinct from their European counterparts (e.g., [19]). Most studies have assumed that these taxonomic inconsistencies had little impact because the North American *D* *. pulex* and *D* *. pulicaria* are sister species [10], a view supported by mtDNA sequence [20] and restriction fragment length polymorphism data [21]: the North American *D* *. pulicaria* (shortly NAPC; Figure 1) and *D* *. pulex* (NAPX) are sister lineages in the same mtDNA clade, the ‘Pulicaria group’ following Colbourne et al. [20], while the European *D* *. pulicaria* (EPC) is a member of a very divergent ‘tenebrosa group’, and the European *D* *. pulex* (EPX) forms a clade on its own (Figure 1).

Gene trees built for nuclear loci rendered NAPX sequences paraphyletic with respect to NAPC sequences [10,22], supporting the close genetic relationships of the North American *D* *. pulex* and *D* *. pulicaria* [10]. No detailed study of sequences of nuclear genes has addressed the relationships with the European populations. The few EPC sequences included in *Rab4* [23] and *Ldh*A and *Ldh*B gene trees [24], constructed primarily for the North American species, were clustered in a clade with the NAPC sequences, suggesting a different relationship than the mtDNA phylogeny (Figure 1), where EPC haplotypes are in a separate clade with *Daphnia* *tenebrosa* (TEN). In the *Rab4* and *Ldh* phylogenies, TEN haplotypes from Canada (no Eurasian sequences were included) were a part of the same clade with the NAPC and EPC haplotypes [23,24]. Clustering analysis of microsatellite data for the same specimens used for *Rab4* sequencing placed TEN genotypes in a group with EPC, but did not provide clear evidence of clustering of NAPC with any other species [23].

*Daphnia* *tenebrosa* is an Arctic species reproducing almost exclusively by obligate parthenogenesis [25], distributed in the Arctic of both North America and Eurasia (Figure 1), and its low mtDNA divergence from EPC might be interpreted as a recent origin of the European populations from an Arctic (e.g. Beringian) ancestor [21], while the European *D* *. pulex* (EPX) is thought to be a long-diverged species with no signatures of gene flow with other species [26]. This would imply a different divergence scenario for the European species than in North America.

The interpretation of Vergilino et al. [23] was that the discordance of the *Rab4* gene tree with the mtDNA phylogeny was likely due to stochastic processes such as lineage sorting of *Rab4*, as opposed to hybridization, which was considered a less likely explanation [23] (but see 24). However, the *Ldh*A and *Ldh*B gene trees of Crease et al. [24] are both congruent with *Rab4* in that the EPC and TEN sequences are clustered with NAPC sequences [24], suggesting that mtDNA is actually the outlier.

We test the hypothesis that the close relationship of the North American and European *D* *. pulicaria* at nuclear loci reflects the true evolutionary relationship among the species, and that it is the mtDNA gene tree that is incongruent with the species tree. If the North American *D* *. pulicaria* is evolutionarily more closely related to the European *D* *. pulicaria* and to *D* *. tenebrosa* than to *D* *. pulex* , it would have important implications for the ecological divergence of *D* *. pulicaria* and *D* *. pulex* . For example, the adaptations in *D* *. pulicaria* may trace their origin to an Arctic ancestor, rather than being the result of divergent selection between the sympatric temperate habitats.

We have sequenced the nuclear loci *Rab4* and *Ldh*A as well as the mtDNA gene *ND5* for *D* *. pulicaria* , *D* *. pulex* and *D* *. tenebrosa* sampled from their distribution ranges in Europe, Asia as well as North America, including from the Arctic islands of Svalbard, Iceland and Greenland. *Rab4* and *Ldh*A are located on genomic scaffolds mapped to different chromosomes [27] and thus provide unlinked markers to independently test the evolutionary hypothesis derived from mtDNA. The *Ldh*A locus has played a central role in the study of the divergence of *D* *. pulicaria* and *D* *. pulex* because the polymorphism at this gene, first detected as two electrophoretic-mobility variants, has been implicated in the adaptive divergence between these species due to strong association of F (fast) allele with lake ( *D* *. pulicaria* ) populations throughout North America [11,24]. A recent study identified two amino acid differences between the polypeptides encoded by F allele versus S allele, although their functional significance has not as yet been demonstrated [24]. Interestingly, allozyme surveys in the Arctic detected F allele in various populations of *D* *. tenebrosa* [25,28], and SF heterozygotes have been reported from *D* *. pulicaria* from mountain lakes in Europe [29]. Shared allozymes between the ‘tenebrosa group’ and ‘Pulicaria group’ were interpreted as an introgression [30], but as yet no DNA sequence has been obtained for F allele from *D* *. tenebrosa* or European *D* *. pulicaria* [24].

We use a Markov chain Monte Carlo method for the multispecies coalescent [31] to infer the species tree for *D* *. pulicaria* and *D* *. pulex* from both Europe and North America based on the sequences of the nuclear loci. The method accounts for discrepancies between the gene trees and for any incomplete lineage sorting, and our results strongly suggest that the North American and European *D* *. pulicaria* are sister lineages derived from a common ancestor much more recently than the divergence of *D* *. pulex* . Simulation experiments show that the discordance of the mtDNA gene tree with the species tree is not due to coalescent stochasticity, and we suggest that it is best reconciled by past replacement of the North American *D* *. pulicaria* ’s mtDNA with that of *D* *. pulex* . We discuss these results in their implications for the geography of speciation and the evolution of adaptive divergence between *D* *. pulicaria* and *D* *. pulex* . 

## Materials and Methods

### DNA sequences

This study presents new data for 301 *Daphnia* . In addition to 189 *D* *. pulicaria* , 52 *D* *. pulex* , 20 *D* *. pulex* × *D* *. pulicaria* hybrids and 30 *D* *. tenebrosa* , we included a small number of individuals from other species in the *D* *. pulex* complex, i.e. two *Daphnia* *arenata* , two *Daphnia* *melanica* and 6 *Daphnia* *middendorffiana* (Table S1), and combined our data with those collected for the same loci by Omilian et al. [22], Vergilino et al. [23] and Crease et al. [24].

Genomic DNA was extracted from samples stored in 95% ethanol using the QIAGEN (Valencia, CA) DNeasy Tissue Kit. To reduce the risk of polymerase chain reaction (PCR) errors, a high fidelity DNA polymerase was used (Easy-A high-fidelity PCR cloning enzyme, Agilent Technologies, CA; LA DNA Polymerases mix, Top-Bio, Prague, Czech Republic). The PCR products were purified with the QIAquick PCR Purification Kit and were directly cycle-sequenced with the ABI Prism BigDye chemistry and a 3730xl DNA analyser (Applied Biosystems, Foster City, MA).

A part of the gene coding the small GTPase *Rab4* (Table 1), including one partial and three complete exons, two introns (an extra intron segregating in some populations was excluded from the analysis, see 22), and a small portion of the 3’ untranslated region (UTR), was amplified and sequenced using previously described primers F6for and F12rev [22]. The entire lactate dehydrogenase A (*Ldh*A) gene, consisting of six exons and five introns, was amplified and sequenced with newly designed forward (5’-AATTTGATTGTCTGCTTGAAT-3’) and reverse primers (5’-CGTGTATTTTACTRGGACAYAAC-3’). A part of the mitochondrial gene *ND5*, encoding the NADH dehydrogenase subunit 5, was amplified with published primers as described by Dufresne et al. [32]. DNA sequences have been deposited in GenBank under the accession nos KC536132-KC536502 (*Rab4*), KC535963-KC536131 (*Ldh*A), and KC536503-KC536623 (*ND5*).

**Table 1:** Loci studied and polymorphism summary.

Locus	Sample size	Alignment length (bp)	Number of haplotypes	Polymorphic sites	Relative substitution rate (95% HPD)
*Rab4*	524	464	83	72	1.00 (N/A)
*LdhA*	309	1273	117	172	1.22 (0.74–1.85)
*ND5*	318	623	133	149	15.17 (9.44–22.62)

Chromatograms were imported in the CodonCode Aligner software (CodonCode Corporation) for base calling, assembly and heterozygote detection. All homozygous *Ldh*A sequences were verified by amplifying and sequencing that individual using a different set of newly designed forward (5’-GCCCAYTCAGGAAGCAAAGTTA-3’) and reverse (5’-AGDACATATTTTATAATACCMAATT-3’) primers as well as with a set of published primers (LDHA-u4F and LDHA-1304R [24]). All *Rab4* and *Ldh*A genotypes containing multiple heterozygous sites were resolved into haplotypes by cloning with the QIAGEN PCR Cloning Plus Kit. Ten to 30 clones were sequenced for each cloned amplicon and the results were compared to the sequences obtained by the direct sequencing to ensure that polymorphisms were not the result of PCR or cloning-induced errors [33].

### Data analyses

The alignments were stripped of gaps and identical sequences collapsed into haplotypes using MacClade 4.08 [34]. Polymorphism and divergence at each locus were summarized using the programs DnaSP, version 5.10 [35], and SITES [36]. In this summary and in the species tree analysis (see below), the *Rab4* and *Ldh*A datasets for the European *D* *. pulicaria* exclude the NAPC sequences from EPC × NAPC heterozygotes (see below) and the sequences from the TEN mtDNA background. Similarly, the datasets for the North American *D* *. pulicaria* exclude *D* *. melanica* and *D* *. middendorffiana* sequences and those sampled from the SAPC and TEN mtDNA backgrounds, but NAPC sequences from the NAPX mtDNA background are included, reflecting the fact that many *D* *. pulicaria* in North America carry NAPX mtDNA [11]. The North American *D* *. pulex* excludes the sequences of *D* *. middendorffiana* and those from the TEN mtDNA background. Only TEN sequences from the TEN mtDNA background were included in *D* *. tenebrosa* , excluding the sequences of EPC and NAPC haplotypes from the TEN mtDNA background.

The alignments of the two nuclear genes were searched for evidence of recombination using a suite of phylogenetic-substitution- and distance-based methods [37] included in the RDP4 software package [38]. The alignments were first searched with the RDP method [39], GENECONV [40] and MAXCHI [41]. In case of a significant signal, it was then further checked with BOOTSCAN [42], SISCAN [43], 3SEQ [44], CHIMAERA [45] and LARD [46]. Analyses were run on full alignments (unique haplotypes only) as well as on reduced alignments where sequences sharing more than 70% identity to other sequences were masked. Comparisons among similar sequences are unlikely to yield detectable signal of recombination and masking similar sequences increases the power of multiple tests. The *P*-value cut-off was set to 0.05 in all analyses and the Bonferroni correction was applied. The analyses settings were kept at defaults except that the RDP and MAXCHI analyses were repeated with the sliding window size set to 10, 30, 15, 100 and 200 alignment sites (variable sites for MAXCHI).

Gene trees were constructed from the haplotype alignments using the maximum likelihood optimality criterion. The program jModelTest 0.1.1 [47] was used to determine the best-fit evolutionary model for each locus based on the Akaike information criterion. The maximum-likelihood phylogenetic analyses were performed by the combination of the NNI (nearest neighbour interchanges) and SPR (subtree pruning and regrafting) searches as implemented in GARLI 2.0 [48], with character partitions according to exon, intron and UTR regions and codon positions in coding regions (Table 2). Multiple GARLI runs were performed for each dataset to ensure convergence on the same topology, each consisting of several replicates (10 for *Rab4* and 5 for *Ldh*A and *ND5*) started with a different random tree topology and with the termination conditions set to 50 000 generations without topology improvement, a 0.00001 increase for a significantly improved topology, and a score improvement threshold of 0.001. Bootstrap support was estimated from 1000 bootstrap samples with five search replicates (one for *ND5*) performed for each bootstrap sample and the termination criterion reduced to 2000 generations and topology improvement and score threshold to 0.01 and 0.05, respectively. Because distant outgroups can have adverse effects on the relationships within the ingroup we rooted the gene trees with the sequences of EPX that other researchers considered outgroup to EPC, NAPC and NAPX (e.g., [19,20]); the EPX root is also the best-supported root in our species tree analyses (see below).

**Table 2:** Best-fit models of sequence evolution for character partitions in each locus.

Locus	1st codon	2nd codon	3rd codon	Intron	3’UTR	Entire gene
*Rab4*	TPM1	HKY	TPM3uf+G	TPM3uf+I	F81	TVM+I+G
*LdhA*	HKY	HKY	TVM+I+G	HKY+G	N/A	GTR+I+G
*ND5*	TPM2uf+I+G	HKY+I	GTR+G	N/A	N/A	TVM+I+G

The species tree for *D* *. pulex* (European and North American) and *D* *. pulicaria* (European and North American) was estimated using the Markov chain Monte Carlo (MCMC) method for the multispecies coalescent implemented in *BEAST [31]. The method infers the species tree from multiple genes sampled from multiple individuals of each species [31]. We did not include *D* *. tenebrosa* because it reproduces almost exclusively asexually throughout its distribution [25,28], violating the *BEAST assumption of recombination between loci [31]. Temperate populations of *D* *. pulex* from western North America and temperate eastern populations of *D* *. pulicaria* reproduce by cyclical parthenogenesis (series of asexual generations interrupted by a sexual generation [49]), and since many of the samples in our study were from these populations, we considered that in these species and on the time scale relevant for the coalescent analysis, the loci can be considered effectively independent.

Because of the suspected incongruity of the mtDNA gene tree with the species tree, we inferred the four-species tree from the nuclear loci (nuclear four-species tree). For comparison, we also estimated a three-species tree including all loci but excluding NAPC as the suspected source of the nuclear-mtDNA incongruity (three-locus three-species tree). Finally, we estimated the four-species tree from mtDNA data only (mtDNA four-species tree). Because *BEAST makes the assumption that there is no gene flow following the species divergence [31], no allospecific haplotypes were included in the input species data sets (see above). We used a GTR+I+G model of sequence evolution for *Ldh*A and *ND5* and GTR+I for *Rab4*, which were the next-highest scoring models that were available, and an uncorrelated log-normal relaxed molecular clock with a separate substitution rate for each locus. We fixed the substitution rate for *Rab4* at 1 and estimated the rates for the other loci relative to this locus. Each analysis was repeated to ensure MCMC convergence, and log and tree files from repeated runs were combined when necessary to give the effective sample sizes of >200. We repeated the analyses also assuming strict molecular clock, and also fixing the substitution rate for *Ldh*A instead of *Rab4*.

To test whether the incongruence between the mtDNA gene tree and the estimated species tree might be due to coalescent stochasticity such as the failure of lineages to coalesce between speciation events (deep coalescence [50]), we performed simulations with the program Mesquite 2.75 [51]. We simulated 10 000 gene trees of 274 gene copies under the species tree inferred from the nuclear loci. The gene trees corresponded to the same sample size as our *ND5* dataset, with the same number of gene copies sampled from each of the four species (Table 3). The estimated divergence times of the species tree were converted to the number of generations assuming a mutation rate of 10^-9^ per site per generation [52], and the simulation was repeated for a range of the effective population sizes, *N*_*e*_, for the individual species (equal for all species), equivalent to 100 000, 500 000, 1 000 000 and 1 500 000, which encompassed the empirical estimates [10,53]. To determine if the observed mtDNA gene tree, with NAPC haplotypes in a clade with NAPX haplotypes, could have been generated under the estimated species tree, we recorded the percentage of the gene trees simulated for a given *N*_*e*_ that had NAPC and NAPX genes as a clade. 

**Table 3:** Genic diversity within species.

	Sample size	Number of Haplotypes	Polymorphic sites	Haplotype diversity ± SD	Nucleotide diversity ± SD
EPC					
*Rab4*	159	7	7	0.264 ± 0.045	0.00063 ± 0.00012
*Ldh*A	50	18	16	0.900 ± 0.025	0.00189 ± 0.00016
*ND5*	144	37	55	0.947 ± 0.008	0.02689 ± 0.00079
NAPC					
*Rab4*	132	15	15	0.865 ± 0.014	0.00764 ± 0.00023
*Ldh*A	89	17	30	0.755 ± 0.033	0.00255 ± 0.00026
*ND5*	47	21	44	0.944 ± 0.016	0.01983 ± 0.00070
NAPX					
*Rab4*	97	30	39	0.954 ± 0.007	0.01793 ± 0.00078
*Ldh*A	84	42	69	0.954 ± 0.013	0.01002 ± 0.00090
*ND5*	36	7	10	0.690 ± 0.057	0.00518 ± 0.00076
EPX					
*Rab4*	42	6	4	0.530 ± 0.082	0.00147 ± 0.00030
*Ldh*A	39	13	19	0.907 ± 0.025	0.00338 ± 0.00030
*ND5*	47	13	23	0.901 ± 0.023	0.01110 ± 0.00092
TEN					
*Rab4*	45	21	35	0.900 ± 0.031	0.01094 ± 0.00108
*Ldh*A	20	17	56	0.984 ± 0.020	0.00851 ± 0.00098
*ND5*	33	16	64	0.941 ± 0.019	0.03028 ± 0.00442

## Results

### Detection of recombination

Two recombination events were detected at *Ldh*A and none at *Rab4*. Only the substitution-based methods MAXCHI and 3SEQ yielded significant result (*P*<0.01 and *P*<0.05, respectively), but they were congruent in that the recombination signals consistently involved the sequence OR-31 ( *D* *. pulex* from Oregon [24]), and masking this sequence yielded no significant signal. Therefore, we considered OR-31 as putative recombinant and performed the phylogenetic and species tree analyses also excluding this sequence.

### Genealogical relationships

**Figure 2:**
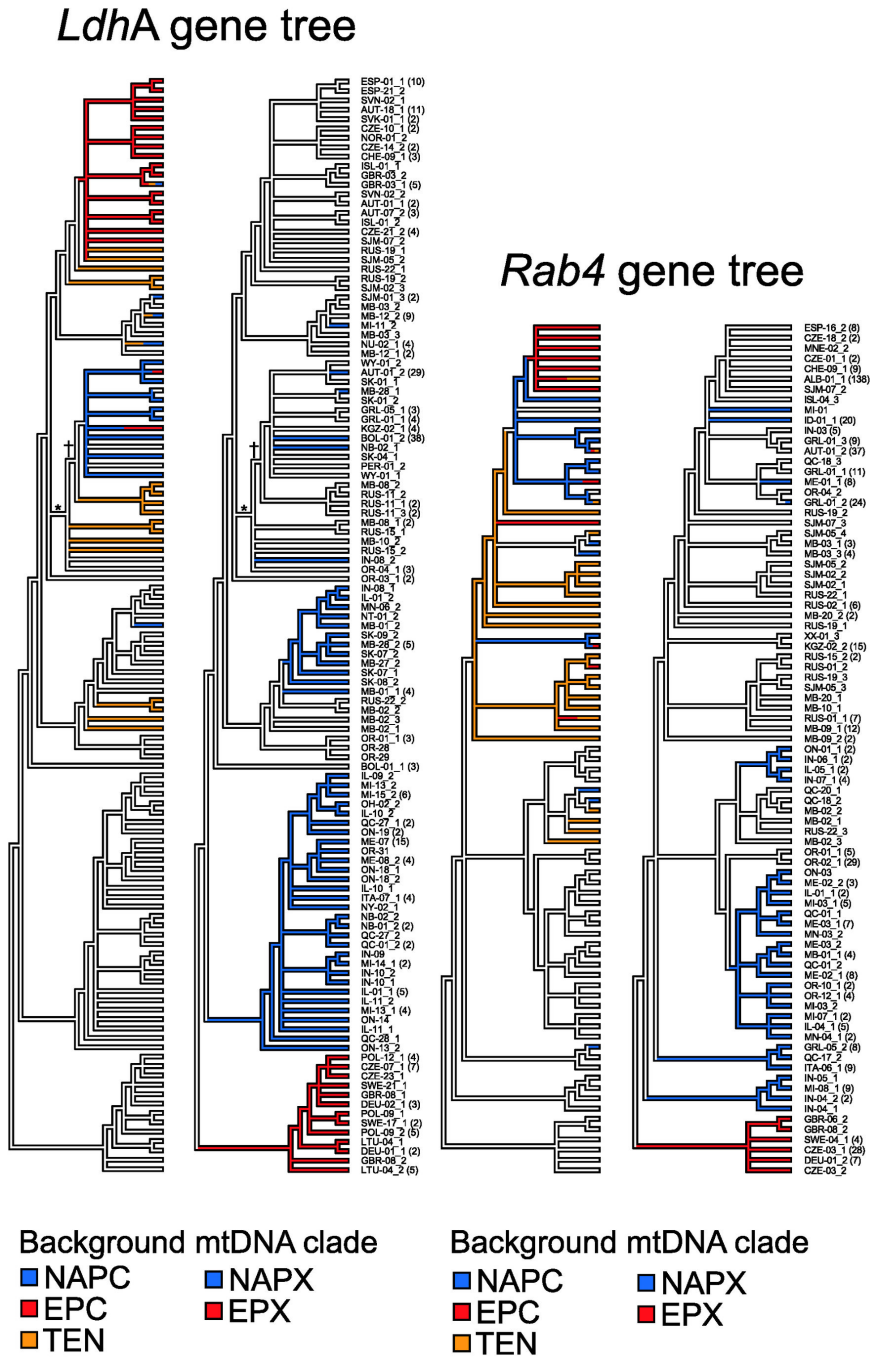
Gene trees for the *Ldh*A and *Rab4* haplotypes. The trees are maximum-likelihood topologies with branches coloured to indicate the mtDNA clades carried by the individuals in which the haplotypes were samples. Clade acronyms are the same as those used in Fig. 1. The two- or three-letter code names correspond to the individuals’ sampling localities (Table S1) and the numbers following the underline character to different alleles within heterozygotes. Only one individual is listed for each haplotype to save space and the number of individuals carrying that haplotype is noted in parentheses when higher than one (for trees showing all individuals, branch length estimates and bootstrap frequencies, see Figs S1 and S2; Supporting Information). Amino acid substitutions distinguishing the pond (S) and lake (F) alleles of *Ldh*A are indicated as follows: *, charge-changing Gln229Glu substitution; †, charge-conservative Asp6Glu substitution.

Although the *Rab4* data contained less variation than the *Ldh*A data (Table 1), resulting in shorter internal branches of the *Rab4* gene tree (Figures S1 and S2), the overall genealogical patterns were remarkably similar in terms of the relationships of the haplotypes sampled from the different mtDNA backgrounds (Figure 2). The exclusion of the *Ldh*A sequence OR-31 had negligible topological effect and we present the results for the full data set only.

**Figure 3:**
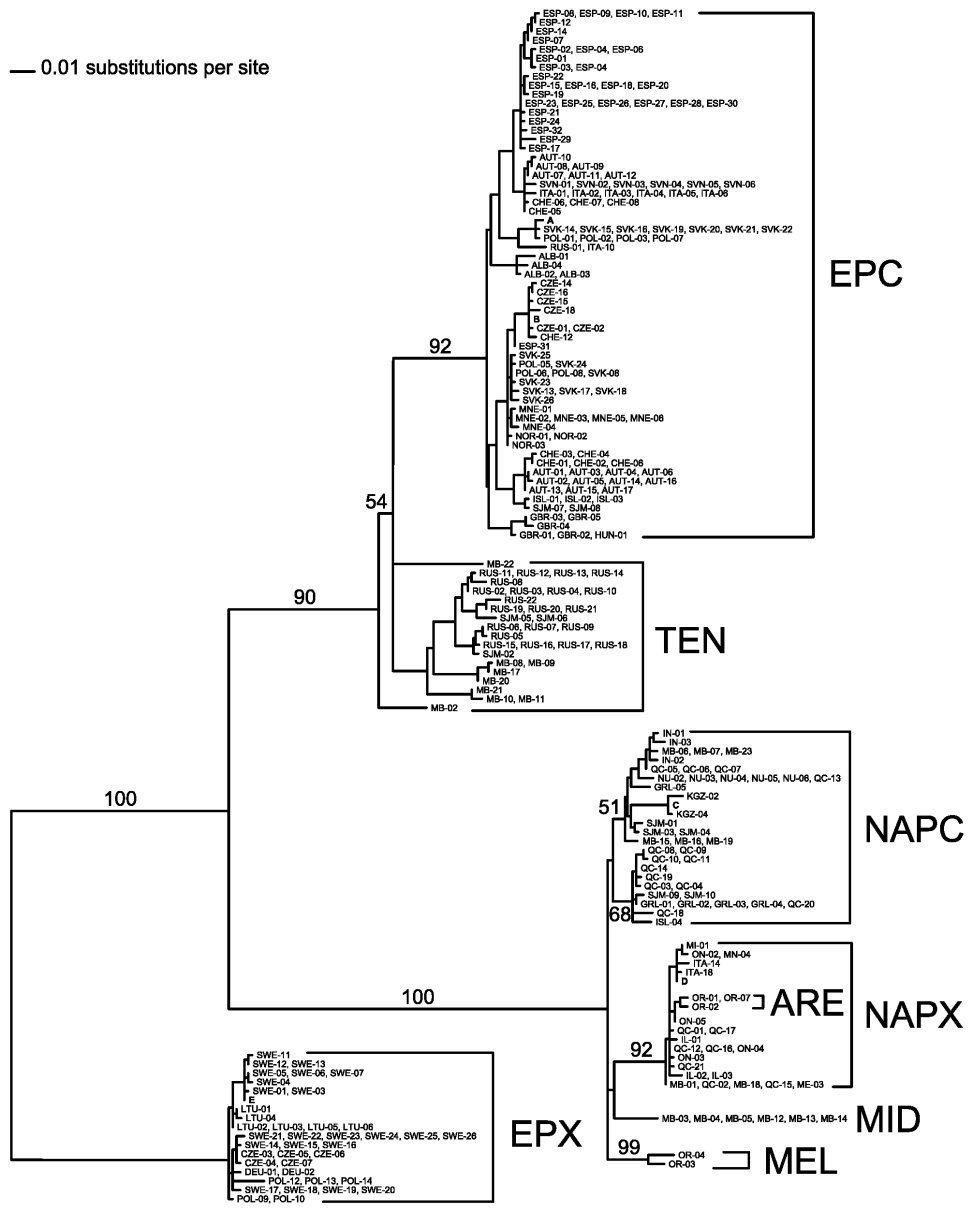
A mitochondrial DNA gene tree inferred by maximum likelihood from the sequences of *ND5* haplotypes. Numbers along branches indicate the percent bootstrap frequencies for major bipartitions. Haplotypes carried by many individuals are represented by a letter as follows: A, SVK-01, SVK-03, SVK-04,SVK-05, SVK-06, SVK-07, SVK-09, SVK-10, SVK-11 and SVK-12; B, CZE-09, CZE-10, CZE-11, CZE-19, CZE-20, POL-11, CHE-09, CHE-10, CHE-11 and DEU-03; C, KGZ-01, KGZ-03, KGZ-05, KGZ-06, KGZ-07 and KGZ-08; D, ITA-06, ITA-07, ITA-08, ITA-09, ITA-11, ITA-12, ITA-13, ITA-15, ITA-16, ITA-17, ITA-19, ITA-20, ITA-21, ITA-22 and ON-01; E, GBR-06, GBR-07, GBR-08, SWE-02, SWE-08, SWE-09 and SWE‑10.

The two nuclear gene trees agree well also with the mtDNA gene tree in many aspects. The EPX haplotypes formed a clade in both *Rab4* and *Ldh*A gene trees (Figure 2) as well as in the mtDNA genealogy (Figure 3). The nuclear haplotypes sampled from the NAPX mtDNA background did not form a single clade in either nuclear gene tree, but there was one major clade in each genealogy collecting only haplotypes sampled from the NAPX mtDNA background (Figure 2), plus there were two smaller NAPX clades in the *Rab4* gene tree. In addition, there was a NAPX clade in the *Rab4* as well as *Ldh*A gene tree that contained a small number of haplotypes sampled from the NAPC mtDNA background and four haplotypes sampled from the TEN mtDNA background (see below).

However, both nuclear genealogies were in stark contrast with the mtDNA gene tree (Figure 3) in that the vast majority of *Ldh*A and *Rab4* haplotypes sampled from the NAPC mtDNA background were consistently placed in a clade with the haplotypes sampled from the EPC mtDNA background. The NAPC mtDNA are however clustered in a highly divergent clade together with the NAPX mtDNA (‘Pulicaria group’; Figure 1) while the EPC mtDNA form a distinct clade with the TEN mtDNA (‘tenebrosa group’), and both these clades have high bootstrap support (Figure 3). These contrasting relationships are reflected by the low *ND5* divergence between NAPC and NAPX and high divergence between EPC and NAPC, while the pattern is essentially reversed at *Rab4* and *Ldh*A (Table 4).

**Table 4:** Divergence between species.

Species 1–species 2	*Rab4*	*Ldh*A	*ND5*
Average divergence per base pair			
EPC–NAPC	0.010	0.008	0.183
EPC–NAPX	0.023	0.019	0.177
EPC–EPX	0.018	0.021	0.179
NAPC–NAPX	0.022	0.017	0.043
NAPC–EPX	0.018	0.019	0.193
NAPX–EPX	0.017	0.019	0.189
Net divergence per base pair^1^			
EPC–NAPC	0.006	0.006	0.159
EPC–NAPX	0.014	0.013	0.161
EPC–EPX	0.017	0.018	0.162
NAPC–NAPX	0.009	0.011	0.030
NAPC–EPX	0.013	0.016	0.178
NAPX–EPX	0.008	0.012	0.182

^1^ Average divergence minus average diversity within each of the two species.

The nuclear genes sampled from the TEN mtDNA background do not form a clade on their own but are clustered in the clade with *D* *. pulicaria* haplotypes where the majority of TEN haplotypes branch off at a basal position relative to the EPC or NAPC haplotypes, irrespective of the geographic origin (Figure 2 Figures S1 and S2). This agrees with the clustering analysis of genetic distances between microsatellite genotypes, which placed TEN clones from Churchill, Manitoba, in a group with EPC genotypes but only one NAPC genotype [23]. The basal position and paraphyly of the TEN haplotypes relative to the *D* *. pulicaria* haplotypes is common to all the three gene trees, except that in the mtDNA gene tree only EPC haplotypes, but not NAPC haplotypes, are placed in the same clade with the TEN haplotypes (Figure 3).

Consistent between both nuclear gene trees, four haplotypes from the TEN mtDNA background, three from an individual from Churchill, and one from an individual from Taimyr Peninsula, Russia, were placed in a clade with the NAPX haplotypes (Figure 2). These results show that some NAPX-like haplotypes are rarely present on the TEN mtDNA background not only in North America [54], but also in Eurasia, although the majority of S-class *Ldh*A haplotypes in *D* *. tenebrosa* are haplotypes related to *D* *. pulicaria* and not *D* *. pulex* (Figure 2).

Consistent with the mtDNA genealogy, *D* *. arenata* haplotypes were clustered with the NAPX haplotypes in both nuclear gene trees, while *D* *. melanica* haplotypes were clustered with the NAPC haplotypes (Figures S1 and S2); in the mtDNA gene tree the MEL haplotypes branch off at the base of the ‘Pulicaria group’ clade (Figure 3). Sequences of NAPC as well as NAPX haplotypes were sampled from the MID mtDNA background, which is consistent with the presumed hybrid origin of *D* *. middendorffiana* clones [55].

### Species tree reconstruction

**Figure 4:**
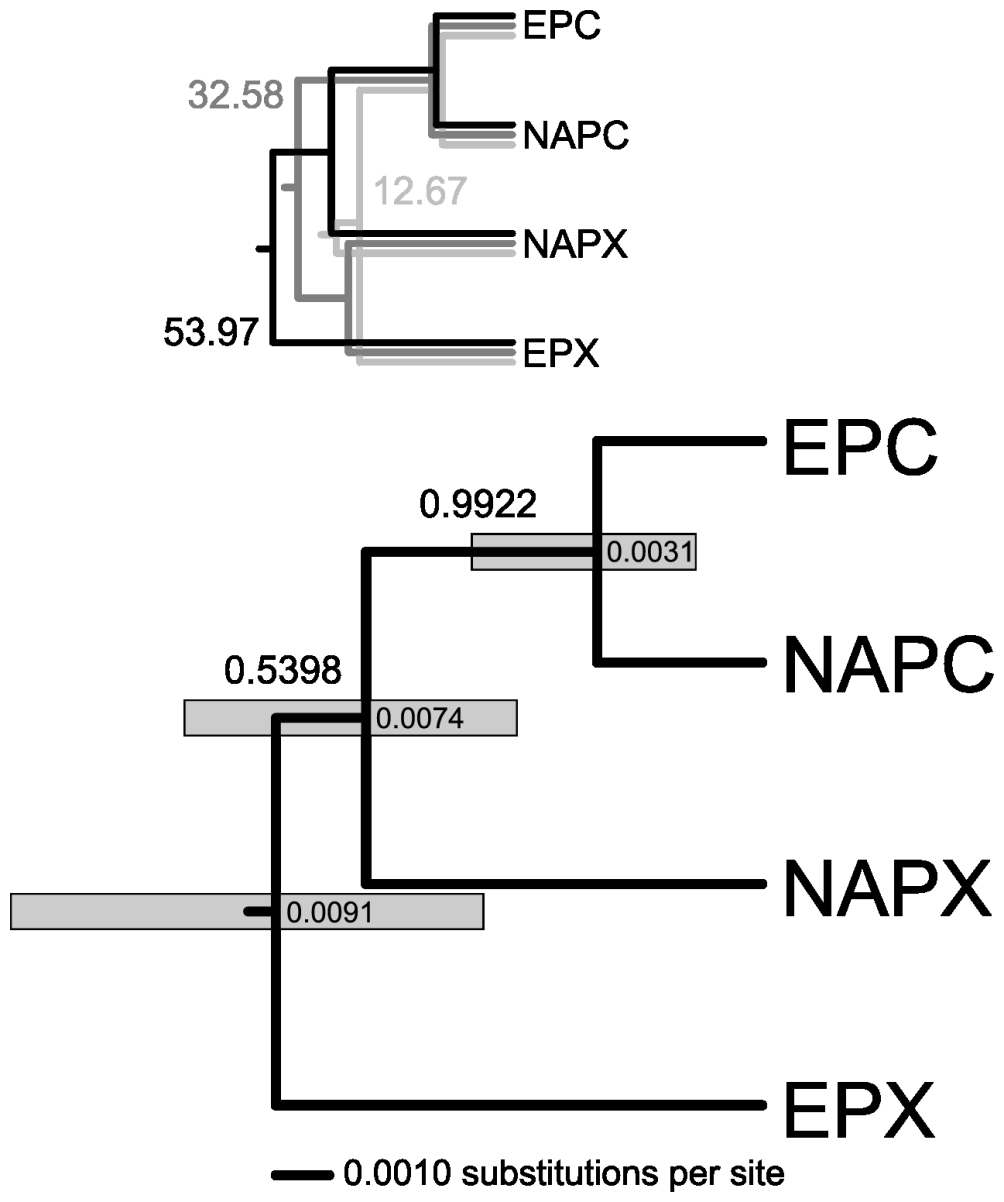
Bayesian species tree. Species tree for the European and North American *Daphnia pulicaria* (EPC and NAPC) and *Daphnia pulex* (EPX and NAPX) inferred from the nuclear gene data by the multispecies coalescent in *BEAST. The tree is a maximum clade credibility tree with clade probabilities indicated above branches. Nodes bars are the 95% highest posterior density intervals for the node ages with median values within the bars. Inset: The 99% credible set of trees containing three topologies with the indicated frequencies.

The *BEAST analysis of the nuclear genes unambiguously recovered the European *D* *. pulicaria* as the sister lineage to the North American *D* *. pulicaria* (Figure 4). The posterior probability of this relationship exceeding 0.99 implies that virtually all species trees in the posterior distribution had *D* *. pulicaria* monophyletic. The maximum clade credibility species tree in Figure 4 has the North American *D* *. pulex* as the sister lineage to the *D* *. pulicaria* clade. Although this was the most frequent species tree topology in the posterior sample, the support for this branching order was not high. Of the three species tree topologies contained in the 99% credible set (Figure 4), two had *D* *. pulex* paraphyletic with respect to *D* *. pulicaria* , differing only in whether the North American (posterior frequency 0.54) or European *D* *. pulex* (0.13) was the sister lineage to *D* *. pulicaria* . The third topology, which was the second most frequent (0.33), had *D* *. pulex* monophyletic. Therefore, although a bit uncertain about the root position, the species tree reconstruction provides a fundamentally different picture of the ancestral relationships between *D* *. pulicaria* and *D* *. pulex* than suggested by the mtDNA gene tree where NAPC and NAPX are sister clades (Figures 1 and 3). A species tree topology with the European *D* *. pulex* root was also the most frequent topology (0.68) in the three-locus three-species tree analysis that included the mtDNA data but excluded the North American *D* *. pulicaria* (results not shown). Consistent with the topology of the mtDNA gene tree, a four-species tree estimated only from the mtDNA data recovered the North American *D* *. pulex* as the sister lineage to the North American *D* *. pulicaria* with the probability 1.00 (results not shown).

Fixing the substitution rate for *Rab4* yielded an estimate of the mean rate for *Ldh*A that was only slightly higher and statistically undistinguishable from 1 (Table 1). The mean rate for *ND5*, estimated in the three-locus three-species analysis, was on the other hand significantly faster, with the mean approximately 15× higher than the rates of the nuclear loci (Table 1). The relative rate estimates were consistent between the analyses fixing the substitution rate for different loci, and all the results were essentially identical in the analyses assuming fixed (results not shown) rather than relaxed clock.

### Coalescent simulation

Virtually none of the gene trees simulated under the inferred species tree to match our sampling effort of the *ND5* sequences had NAPC and NAPX genes as a clade when assuming the species’ *N*_*e*_ equal to 100 000 and to 500 000. Even in the simulations assuming *N*_*e*_ as high as 1 000 000 and 1 500 000 (an upper bound of the empirical estimate [10]), the probabilities of observing a gene tree with NAPC and NAPX forming a clade exclusive to EPC were *P*<0.01 and *P*<0.05, respectively. Instead, over 95% of gene trees in each simulated set had *D* *. pulicaria* genes as a monophyletic group, showing that the incongruous mtDNA gene tree unlikely is due to coalescent stochasticity. 

## Discussion

The North American *D* *. pulicaria* and North American *D* *. pulex* are thought to have originated by adaptation to ecologically distinct but geographically overlapping habitats [10,11]. Their sister-clade mtDNA relationship [20] and the absence of reciprocal monophyly at nuclear genes were considered as the evidence that the two species began to diverge from the common ancestor relatively recently [10,11]. Our Bayesian species-tree reconstruction based on nuclear loci and including large samples of *D* *. pulex* and *D* *. pulicaria* from both Europe and North America provided a very different view, however, as it resolved the European *D* *. pulicaria* as the sister lineage to the North American *D* *. pulicaria* (Figure 4).

The multispecies coalescent implemented in *BEAST accounts for incomplete lineage sorting. Indeed, the gene trees estimated for the nuclear loci failed to recover the EPC, NAPC and NAPX haplotypes as monophyletic clades (Figure 2), showing that the gene lineages have not yet sorted within these species. Therefore, if the mtDNA phylogeny matched the true species tree and the discordant nuclear gene trees were due to incomplete lineage sorting [23], *BEAST should have correctly recovered the North American *D* *. pulex* and *D* *. pulicaria* as sister species. However, the *D* *. pulicaria* clade had very high statistical support in the *BEAST analyses, with virtually all species trees in the posterior sample having the European and North American *D* *. pulicaria* as sister lineages (Figure 4). Excluding the North American *D* *. pulicaria* but including mtDNA resulted in the topology that was compatible with the topology of the four-species tree based on the nuclear loci only, suggesting that the nuclear loci and mtDNA support the same species-tree hypothesis when the North American *D* *. pulicaria* is excluded. Our results thus strongly suggest that it is the mtDNA gene tree that is incongruent with the species tree in the *D* *. pulex* complex and that the incongruence is due to the NAPC mtDNA being closely related to NAPX mtDNA while the sister species to the North American *D* *. pulicaria* is the European *D* *. pulicaria* .

**Figure 5:**
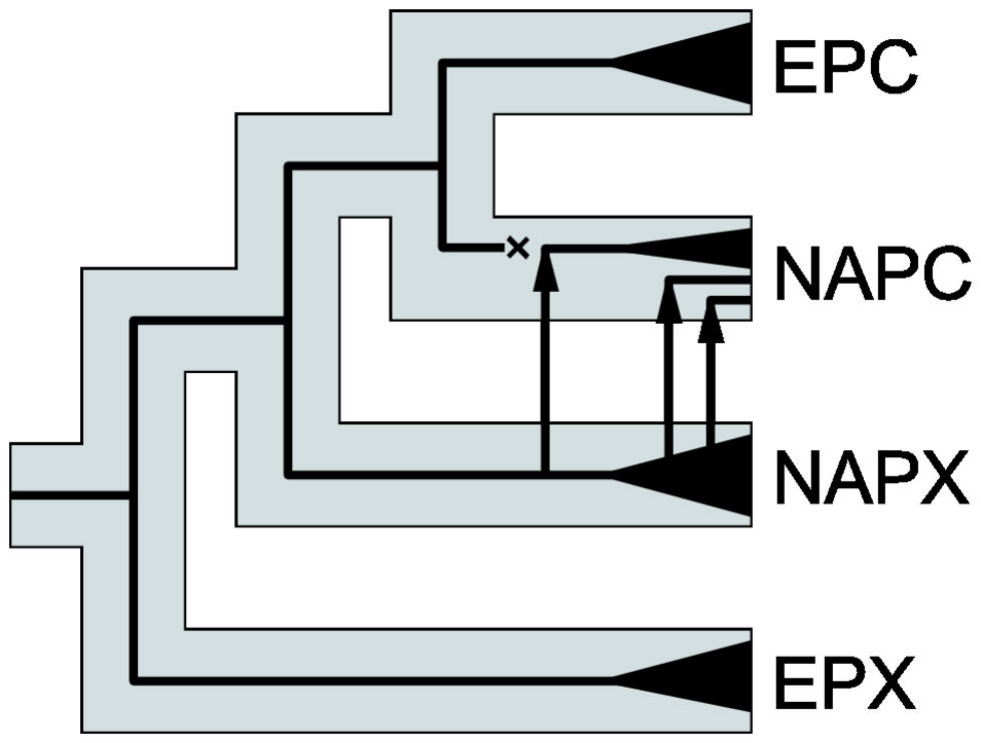
Schematic scenario reconciling the discordance of the mtDNA gene tree with the species tree. Grey bars represent the species tree, thick black lines the mtDNA gene tree, and black wedges denote mtDNA clades within each species. The cross symbolises the hypothesized disappearance of the autochthonous *Daphnia pulicaria*’s mtDNA in North America due to its replacement with that of *Daphnia pulex*. The arrows indicate the direction of this ancient mtDNA introgression as well as of a more recent introgression of the haplotypes from the NAPX clade.

The simulation experiments strongly suggest that the discordance of the mtDNA gene tree with the species tree is not due to coalescent stochasticity, e.g. deep coalescence. Given the propensity of the North American *D* *. pulicaria* to hybridize with *D* *. pulex* , the incongruous mtDNA gene tree is thus best explained by an introgression of *D* *. pulex* mtDNA into *D* *. pulicaria* (Figure 5). The autochthonous *D* *. pulicaria* ’s mtDNA appears to have been completely replaced throughout North America as no haplotypes from the ‘tenebrosa group’ were found in the temperate regions and those found in the Arctic Canada and Alaska were all TEN haplotypes [21,25,56] (Figure 1). Introgression of allospecific mtDNA through hybridization, which may proceed towards complete replacement of the autochthonous mtDNA (mtDNA capture), has been recognized as the source of incongruence between mtDNA and species trees in other organisms (e.g., [57,58]; for a review see 59). In *D* *. pulicaria* , the NAPC haplotypes form a distinct clade from, although closely related to, the NAPX clade, suggesting that the introgression and replacement of the original *D* *. pulicaria* ’s mtDNA is not a recent event. The scaled divergence time of the species trees estimated from mtDNA suggests that the time of the introgression (0.0013 substitutions per site ago) is approximately 40% of the time since the separation of the North American and European *D* *. pulicaria* (0.003 substitutions per site; Figure 4). Previous studies have attempted to date the divergence between *D* *. pulicaria* and *D* *. pulex* on the absolute time scale, yielding estimates ranging from several million years when estimated from mtDNA genetic distance [20] to tens of thousands years when based on nuclear loci and a coalescent model with migration [10]. Notably, the published estimate from mtDNA is an order of magnitude higher that that based on nuclear loci, while our introgression scenario necessarily predicts a lower divergence time between NAPC and NAPX mtDNA than the actual species divergence between *D* *. pulicaria* and *D* *. pulex* (Figure 5). Our *BEAST analyses reconciled the differences in the evolutionary rates between the two nuclear loci and *ND5* by estimating a 15× higher substitution rate per site for *ND5* than for *Rab4* and *Ldh*A, which is consistent with the approximately 10× faster mtDNA than nuclear mutation rate experimentally determined for *D* *. pulex* [52]. Therefore, the discrepancy between the published divergence time estimates can most likely be attributed to the different methodologies, mutation rates and/or datasets used in those studies.

It is remarkable that mtDNA of *D* *. pulex* continues to introgress into *D* *. pulicaria* , as many *D* *. pulicaria* today carry NAPX haplotypes [11,23]. It has been suggested that the unidirectional introgression of mtDNA from pond ( *D* *. pulex* ) populations into lake ( *D* *. pulicaria* ) populations is the result of a combination of natural selection against immigrants and of asymmetric hybridization, when *D* *. pulicaria* occasionally disperse into ponds where the males produced by the dispersers backcross to abundant resident females [11]. We suggest that this scenario explains the capture of *D* *. pulex* mtDNA by *D* *. pulicaria* . An action of selection has been invoked to explain mtDNA capture in other species [59], and it possible that in the temperate regions of North America, the *D* *. pulex* mtDNA conveys a selective advantage over *D* *. pulicaria* mtDNA. Under such scenario, an ancient selective sweep of *D* *. pulex* mtDNA through *D* *. pulicaria* would have been followed by the appearance of new variation in *D* *. pulex* (NAPX clade) that later introgressed into *D* *. pulicaria* (see 60,61).

The nuclear gene trees agreed with each other and with the mtDNA gene tree in the placement of the *D* *. tenebrosa* haplotypes, which branched off basal to *D* *. pulicaria* haplotypes (EPC or NAPC) in all the three gene trees. *Daphnia* *tenebrosa* is genetically highly variable relative to the other species (Table 3), which suggests a comparably high long-term effective population size. Some variation might have originated from introgressive hybridization (before the transition to asexuality or via rare sexual reproduction), but we propose that a large number of clones (often polyploid [62]) occurring across vast expanses of the Holarctic slow down the lineage sorting and yields paraphyletic genealogies with respect to *D* *. pulicaria* (Figure 2). Our results therefore support the scenario that the ancestry of the ‘tenebrosa group’ traces back to an Arctic ancestor [21], but they make the North American *D* *. pulicaria* a part of the story, showing that not only the European *D* *. pulicaria* but also the North American *D* *. pulicaria* are likely derived from the same ancestor.

That the North American *D* *. pulicaria* shares a common ancestry with the European *D* *. pulicaria* and with *D* *. tenebrosa* has implications for the origin of the adaptive divergence between lake and pond populations. The *Ldh*A F allele was suspected to be involved in lake adaptation in North America because it is fixed or nearly so in *D* *. pulicaria* while *D* *. pulex* possess the S allele [24,63]. On the other hand, adaptation to the pond habitat is thought to have driven to fixation the S allele in ponds colonized from a polymorphic lake source [64]. The greater anodal mobility of the F allele relative to S allele likely is due to the substitution of the neutral glutamine at the position 229 in the gene’s fourth exon for the negatively charged glutamic acid, plus there is a charge-conservative aspartic to glutamic acid substitution at the position 6 in the first exon in the F allele, although there is as yet no evidence for functional significance of either substitution [24]. As expected, the majority of the *Ldh*A haplotypes that we sampled from *D* *. pulicaria* in North America grouped in the 229Glu (F) haplotype clade, while *D* *. pulex* haplotypes and the majority of the *D* *. pulicaria* haplotypes from Europe were 229Gln (S) haplotypes (Figure 2). However, six *D* *. pulicaria* from Europe (Alps, High Tatra and Norway) were Gln229Glu heterozygotes and two from Central Asia (Kyrgyzstan) carried only 229Glu alleles as did a number of *D* *. tenebrosa* from both North America and Eurasia. Nearly half of the TEN haplotypes were 229Glu (F) haplotypes (Figure 2). Except for Svalbard, where all TEN haplotypes were 229Gln haplotypes, the *D* *. tenebrosa* populations in Europe (Petchora River, Russia), Asia (Taimyr Peninsula, Russia) and North America (Churchill, Manitoba) all contained 229Glu haplotypes. This is in good agreement with earlier allozyme surveys, which found S as well as F allele in *D* *. tenebrosa* across the Holarctic [25,28]. Shared allozymes between the ‘Pulicaria group’ and ‘tenebrosa group’ were interpreted as an introgression, considered frequent throughout the Holarctic [30]. It is however clear from our results that *D* *. pulicaria* and *D* *. tenebrosa* share the Gln229Glu substitution due to common descent and not to introgression, as the TEN 229Glu haplotypes are basal in the 229Glu clade and distinct from the *D* *. pulicaria* 229Glu haplotypes (Figure 2). Interestingly, only four out of eight 229Glu alleles in *D* *. tenebrosa* have the derived amino acid (glutamic acid) at the position 6 (Figure 2). *Daphnia* *tenebrosa* thus segregates the ancestral and derived states at both amino acid sites distinguishing the pond and lake alleles. This is likely due to ancestral polymorphism in the *D* *. pulicaria* clade, although some TEN clones might carry introgressed *D* *. pulex* (S) haplotypes [54] (Figure 2). These results imply that *D* *. pulicaria* acquired the derived states at both sites by inheritance from the ancestor shared with *D* *. tenebrosa* . This does not exclude the possibility that one or both substitutions are adaptive in lake environment [24], but if they are, the adaptation is likely inherited from the *D* *. tenebrosa* ancestry. It remains yet to be determined whether the presence of F allele in *D* *. pulicaria* in Eurasia is due to retention of the ancestral polymorphism or to gene flow from North America. The fact that all *D* *. pulicaria* 229Glu haplotypes found in Eurasia were also sampled in North America, and that NAPC mtDNA also occurs in Eurasia, support the gene flow scenario [65].

Overall, our results suggest that divergent selection between the temperate ponds and lakes likely has not triggered the divergence of *D* *. pulicaria* and *D* *. pulex* . Rather than adaptations to different ecological pressures in their current habitats, the habitat segregation throughout North America might be the consequence of inherited ancestral life-history traits. This would not exclude ecologically-based selection as the driving force of the evolution of the adaptations, but it would mean they have evolved to solve the problems posed by ancestral selection pressures rather than by the current habitats. That *D* *. pulicaria* shares its ancestry with *D* *. tenebrosa* , and that *D* *. tenebrosa* segregates the ancestral and derived states for the amino-acid sites distinguishing the lake and pond alleles suggests that the variation of the Arctic populations may provide important clues about the origin of the adaptive divergence between *D* *. pulicaria* and *D* *. pulex* .

Although theoretical models have deemed sympatric ecological speciation plausible [3,4], compelling examples are scarce and evidence often controversial [7–9,66]. Speciation by divergent selection between sympatric habitats was considered a parsimonious scenario to explain the distribution and ecological divergence of *D* *. pulex* and *D* *. pulicaria* in North America. We have demonstrated that the species are not sister clades and therefore fail to satisfy one of the key criteria of sympatric speciation [9]. We have also shown that the suspected adaptation allele has arisen in an ancestor shared by several different species. Our study thus demonstrates the importance of broad geographic and taxon sampling in the evaluation of hypotheses concerning the geography of speciation and inferring the origin of adaptive divergence between sympatric species. 

## Supporting Information

Figure S1 Phylogenetic relationships of the *Ldh*A haplotypes inferred by maximum likelihood. Numbers along branches indicate the percent bootstrap frequencies for bipartitions with greater than 70% support. To save space, haplotypes carried by many individuals are represented by a letter as follows: **A**, ESP-01_1, ESP-20_1, ESP-20_2, ESP-21_1, ESP-26_1, ESP-26_2, ESP-27_1, ESP-27_2, ESP-28_1, ESP-28_2; **B**, AUT-18_1, CHE-06_1, CZE-14_1, CZE-21_1, CZE-22, ESP-01_2, ESP-33, GBR-01_1, GBR-01_2, ISL-01_3, ITA-01_1; **C**, AUT-01_2, CHE-01_2, ID-02_2, IL-06, IL-07, IL-08, IN-01_1, IN-01_2, IN-02_1, IN-02_2, MB-24_2, MB-25_1, MI-10, NB-03_1, NOR-01_1, ON-13_1, ON-10, PA-02, QC-03_1, QC-03_2, QC-09_1, QC-09_2, QC-12_1, QC-26_1, SK-05_1, SK-06_1, SVK-26_2, SVN-02_3, WA-02; **D**, BOL-01_2, BOL-02_2, GRL-01_2, GRL-01_3, GRL-03_1, GRL-03_2, GRL-04_1, GRL-04_2, ID-02_1, ITA-07_2, ITA-18_2, KGZ-03_2, MB-06_1, MB-06_2, MB-12_3, MB-24_1, MB-25_2, MB-27_1, ME-05_1, ME-06_1, MI-11_1, MI-12_1, NB-01_1, NU-02_2, NT-01_1, ON-11_1, ON-12_1, QC-02_1, QC-05_1, QC-05_2, QC-25_1, QC-25_2, SJM-09_2, SJM-09_3, SK-02, SK-03_1, SK-03_2, SK-04; **E**, MB-28_2, SK-05_2, SK-06_2, SK-08_1, SK-09_1; **F**, MI-15_2, MI-16_2, NY-03, ON-16, SK-06_2, WI-04_1; **G**, ME-07, ME-08_1, MI-17_2, MI-18, MN-06_1, ON-02_1, ON-02_2, ON-11_2, ON-12_2, ON-15, QC-01_1, QC-28_2, QC-29, QC-30_1, WI-03_2.
(EPS)

Figure S2 Phylogenetic relationships of the *Rab4* haplotypes inferred by maximum likelihood. Numbers along branches indicate the percent bootstrap frequencies for bipartitions with greater than 70% support. To save space, haplotypes carried by many individuals are represented by a letter as follows: **A**, ESP-16_2, ESP-20_2, ESP-22_1, ESP-22_2, GBR-03_1, GBR-03_2, SVK-26_1, SVK-26_2; **B**, CHE-09_1, CZE-09_2, CZE-16_2, CZE-12_2, CZE-13_1, CZE-19, CZE-20, GBR-02_2, POL-11_1; **C**, ALB-01_1, ALB-01_2, ALB-02_1, ALB-02_2, ALB-03_1, ALB-03_2, ALB-04_1, ALB-04_2, AUT-01_1, AUT-02_1, AUT-03_1, AUT-04_1, AUT-05_1, AUT-07_1, AUT-07_2, AUT-08_1, AUT-08_2, AUT-09_1, AUT-09_2, AUT-10_1, AUT-10_2, AUT-11_1, AUT-11_2, AUT-13_1, AUT-14_1, AUT-15_1, AUT-16_1, AUT-17_1, CHE-01_1, CHE-02_1, CHE-03_1, CHE-04_1, CHE-05_1, CHE-05_2, CHE-06_1, CHE-06_2, CHE-09_2, CZE-01_2, CZE-02_2, CZE-09_1, CZE-14_1, CZE-14_2, CZE-16_1, CZE-17_1, CZE-17_2, CZE-18_1, CZE-12_1, CZE-13_2, ESP-01_1, ESP-01_2, ESP-02_1, ESP-02_2, ESP-03_1, ESP-03_2, ESP-04_1, ESP-04_2, ESP-05_1, ESP-05_2, ESP-07_1, ESP-07_2, ESP-08_1, ESP-08_2, ESP-09_1, ESP-09_2, ESP-10_1, ESP-10_2, ESP-11_1, ESP-11_2, ESP-13_1, ESP-13_2, ESP-14_1, ESP-14_2, ESP-15_1, ESP-15_2, ESP-16_1, ESP-18_1, ESP-18_2, ESP-20_1, ESP-23_1, ESP-23_2, ESP-24_1, ESP-24_2, ESP-25_1, ESP-25_2, ESP-27_1, ESP-27_2, ESP-28_1, ESP-28_2, ESP-32, GBR-01_1, GBR-01_2, GBR-02_1, ITA-01_1, ITA-01_2, ITA-02_1, ITA-02_2, ITA-03_1, ITA-03_2, ITA-04_1, ITA-04_2, ITA-05_1, ITA-05_2, ISL-01_1, ISL-01_2, MNE-01_1, MNE-01_2, MNE-02_1, MNE-03_1, MNE-03_2, MNE-04_1, MNE-04_2, MNE-05_1, MNE-05_2, NOR-01_2, NOR-02_2, NOR-03_2, POL-01_2, POL-02_2, POL-04_2, RUS-22_2, SJM-05_1, SJM-07_1, SVN-01_1, SVN-02_1, SVN-03_1, SVN-04_1, SVN-05_1, SVK-01_2, SVK-02_2, SVK-06_2, SVK-07_2, SVK-08_1, SVK-09_2, SVK-14_2, SVK-19_2, SVK-23_1, SVK-25_1, SVK-26_1; **D**, ID-01_1, ID-01_2, IL-03, KGZ-02_1, KGZ-03_1, KGZ-05_1, MI-04_1, MI-04_2, OR-22_1, OR-22_2, QC-02_1, PA-01_2, QC-02_2, QC-09_2, QC-05_1, QC-05_2, QC-12_1, QC-12_2, QC-15, QC-16; **E**, GRL-01_3, GRL-03_3, GRL-04_3, GRL-05_1, MB-19, SJM-01_3, SJM-03_3, SJM-09_3, WA-01_1; **F**, AUT-01_2, AUT-02_2, AUT-03_2, AUT-04_2, AUT-05_2, AUT-13_2, AUT-14_2, AUT-15_2, AUT-16_2, AUT-17_2, CHE-01_2, CHE-02_2, CHE-03_2, CHE-04_2, IL-02, IN-02_1, MB-12_1, MB-22, MI-02_1, MI-02_2, NU-01_1, NU-02_1, OR-03_1, OR-03_2, QC-03_2, QC-20_2, SVN-01_2, SVN-02_2, SVN-03_2, SVN-04_2, SVN-05_2, SJM-01_2, SJM-03_2, SVK-08_2, SVK-23_2, SVK-25_2, SVK-26_2; **G**, GRL-01_1, GRL-03_1, GRL-04_1, ISL-04_2, ME-01_2, QC-09_1, QC-14_1, QC-14_2, QC-18_1, QC-19, SJM-09_1; **H**, ME-01_1, NOR-01_1, NOR-02_1, NOR-03_1, QC-03_1, QC-21, WI-02_1, WI-02_2; **I**, GRL-01_2, GRL-03_2, GRL-04_2, IN-01_1, IN-01_2, IN-02_2, ITA-06_2, ITA-14_2, ITA-15_2, ITA-18_2, ITA-19_2, OR-04_1, MB-06_1, MB-06_2, MB-12_2, MB-23_2, ON-01_2, OR-20_1, ON-07_2, ON-05_1, PA-01_1, QC-17_1, SJM-02_3, SJM-09_2; **J**, KGZ-02_2, KGZ-03_2, KGZ-05_2, MB-16_2, POL-01_1, POL-02_1, POL-04_1, SVK-02_1, SVK-06_1, SVK-07_1, SVK-09_1, SVK-14_1, SVK-19_1, WA-01_2; **K**, MB-09_1, MB-10_2, MB-17_1, MB-17_2, RUS-02_3, RUS-08_3, RUS-11_3, RUS-12_3, RUS-13_3, RUS-14_3, RUS-15_1, RUS-17_1; **L**, OR-02_1, OR-02_2, OR-05_1, OR-05_2, OR-06_1, OR-06_2, OR-07_1, OR-08_1, OR-08_2, OR-09_1, OR-09_2, OR-16_1, OR-16_2, OR-17_1, OR-17_2, OR-18_1, OR-18_2, OR-19_1, OR-19_2, OR-21_1, OR-21_2, OR-23_1, OR-23_2, OR-24_1, OR-24_2, OR-25_1, OR-25_2, OR-26_1, OR-32_1; **M**, ME-03_1, MI-05_1, MI-05_2, ON-01_2, ON-06_2, ON-08_1, ON-09_1; **N**, ME-02_1, NY-01_2, QC-22_1, QC-22_2, QC-23_2, QC-24_1, QC-24_2, WI-01_2; **O**, CZE-03_1, CZE-07_2, CZE-08_1, CZE-08_2, DEU-01_1, GBR-06_1, GBR-07_1, GBR-07_2, GBR-08_1, LTU-01_1, LTU-01_2, LTU-04_1, LTU-04_2, SWE-01_1, SWE-01_2, SWE-08_1, SWE-08_2, SWE-11_1, SWE-11_2, SWE-14_1, SWE-14_2, SWE-17_1, SWE-17_2, SWE-21_1, SWE-21_2, SWE-24_2, SWE-24_1.
(EPS)

Table S1 **Individuals of the *Daphnia* *pulex* species complex included in this study.**
(PDF)
