# Supplementary material for: Mitochondrial Capture Misleads about Ecological Speciation in the Daphnia pulex Complex
Source: PLoS One. 2013 Jul 15;8(7):e69497. doi: 10.1371/journal.pone.0069497 (PMC3711805; doi:10.1371/journal.pone.0069497)
Supplement: Table S1 — Individuals of the Daphnia pulex species complex included in this study. (PDF) [file pone.0069497.s003.pdf]

Table S1. Individuals of the *Daphnia pulex* species complex included in this study

Code	Rab4 GenBank Accession	LDHA GenBank Accession	ND5 GenBank Accession	Species	mtDNA lineage	Latitude	Longitude	Locality	Country	Reference	Lab Key
ALB-01	KC536321, KC536322		KC536551	*D. pulicaria*	EPC	40.95	20.71	Ohrid	Albania	this paper	DP074_004
ALB-02	KC536323, KC536324		KC536552	*D. pulicaria*	EPC	40.95	20.71	Ohrid	Albania	this paper	DP074_005
ALB-03	KC536325, KC536326		KC536552	*D. pulicaria*	EPC	40.95	20.71	Ohrid	Albania	this paper	DP075_004
ALB-04	KC536327, KC536328		KC536553	*D. pulicaria*	EPC	40.95	20.71	Ohrid	Albania	this paper	DP075_005
AUT-01	KC536237, KC536238	KC535985, KC535986	KC536529	*D. pulicaria*	EPC	47.10	11.74	Alps	Austria	this paper	DP036_001
AUT-02	KC536239, KC536240		KC536530	*D. pulicaria*	EPC	47.10	11.74	Alps	Austria	this paper	DP036_005
AUT-03	KC536241, KC536242		KC536529	*D. pulicaria*	EPC	47.10	11.74	Alps	Austria	this paper	DP036_006
AUT-04	KC536243, KC536244		KC536529	*D. pulicaria*	EPC	47.10	11.74	Alps	Austria	this paper	DP036_007
AUT-05	KC536245, KC536246		KC536530	*D. pulicaria*	EPC	47.10	11.74	Alps	Austria	this paper	DP036_008
AUT-06			KC536529	*D. pulicaria*	EPC	47.10	11.74	Alps	Austria	this paper	DP036_009
AUT-07	KC536247, KC536248		KC536531	*D. pulicaria*	EPC	47.04	10.89	Alps	Austria	this paper	DP037_001
AUT-08	KC536249, KC536250		KC536532	*D. pulicaria*	EPC	47.04	10.89	Alps	Austria	this paper	DP037_005
AUT-09	KC536251, KC536252		KC536532	*D. pulicaria*	EPC	47.04	10.89	Alps	Austria	this paper	DP037_006
AUT-10	KC536253, KC536254		KC536533	*D. pulicaria*	EPC	47.04	10.89	Alps	Austria	this paper	DP037_007
AUT-11	KC536255, KC536256		KC536531	*D. pulicaria*	EPC	47.04	10.89	Alps	Austria	this paper	DP037_008
AUT-12			KC536531	*D. pulicaria*	EPC	47.04	10.89	Alps	Austria	this paper	DP037_009
AUT-13	KC536257, KC536258		KC536534	*D. pulicaria*	EPC	46.97	12.28	Alps	Austria	this paper	DP038_001
AUT-14	KC536259, KC536260		KC536530	*D. pulicaria*	EPC	46.97	12.28	Alps	Austria	this paper	DP038_005
AUT-15	KC536261, KC536262		KC536534	*D. pulicaria*	EPC	46.97	12.28	Alps	Austria	this paper	DP038_006
AUT-16	KC536263, KC536264		KC536530	*D. pulicaria*	EPC	46.97	12.28	Alps	Austria	this paper	DP038_007
AUT-17	KC536265, KC536266		KC536534	*D. pulicaria*	EPC	46.97	12.28	Alps	Austria	this paper	DP038_008
AUT-18		KC535987, KC535988		*D. pulicaria*	missing	47.04	10.89	Alps	Austria	this paper	DP037_002
BOL-01		JN117749, JN117740		South American *D. pulicaria*	SAPC	-17.04	-66.61	Ayopaya	Bolivia	Crease et al. 2011	SAPC01
BOL-02		JN117749, JN117740		South American *D. pulicaria*	SAPC	-15.80	-69.38	Titicaca Lake	Bolivia	Crease et al. 2011	SAPC03
CHE-01	KC536277, KC536278		KC536536	*D. pulicaria*	EPC	46.67	8.04	Alps	Switzerland	this paper	DP041_001
CHE-02	KC536279, KC536280		KC536536	*D. pulicaria*	EPC	46.67	8.04	Alps	Switzerland	this paper	DP041_005
CHE-03	KC536281, KC536282		KC536537	*D. pulicaria*	EPC	46.67	8.04	Alps	Switzerland	this paper	DP041_006
CHE-04	KC536283, KC536284		KC536537	*D. pulicaria*	EPC	46.67	8.04	Alps	Switzerland	this paper	DP041_007
CHE-05	KC536285, KC536286		KC536538	*D. pulicaria*	EPC	46.67	8.04	Alps	Switzerland	this paper	DP041_008
CHE-06			KC536536	*D. pulicaria*	EPC	46.67	8.04	Alps	Switzerland	this paper	DP041_009
CHE-06	KC536387, KC536388	KC536019, KC536020	KC536578	*D. pulicaria*	EPC	46.38	7.46	Alps	Switzerland	this paper	DP126_001
CHE-07			KC536578	*D. pulicaria*	EPC	46.38	7.46	Alps	Switzerland	this paper	DP126_002
CHE-08			KC536578	*D. pulicaria*	EPC	46.38	7.46	Alps	Switzerland	this paper	DP126_003
CHE-09	KC536429, KC536430	KC536058, KC536059	KC536546	*D. pulicaria*	EPC	47.70	9.02	Lake Constance	Switzerland	this paper	DP155_001
CHE-10			KC536546	*D. pulicaria*	EPC	47.70	9.02	Lake Constance	Switzerland	this paper	DP155_002
CHE-11			KC536546	*D. pulicaria*	EPC	47.70	9.02	Lake Constance	Switzerland	this paper	DP155_003
CHE-12			KC536591	*D. pulicaria*	EPC	47.70	9.02	Lake Constance	Switzerland	this paper	DP155_004
CZE-01	KC536297, KC536298		KC536543	*D. pulicaria*	EPC	49.78	15.23	Bohdaneč	Czech Republic	this paper	DP048_001
CZE-02	KC536299, KC536300		KC536543	*D. pulicaria*	EPC	49.78	15.23	Bohdaneč	Czech Republic	this paper	DP048_003
CZE-03	KC536301, KC536302		KC536544	*D. pulex*	EPX	49.42	13.78	Blatná	Czech Republic	this paper	DP056_001
CZE-04			KC536545	*D. pulex*	EPX	49.42	13.78	Blatná	Czech Republic	this paper	DP056_002
CZE-05			KC536544	*D. pulex*	EPX	49.42	13.78	Blatná	Czech Republic	this paper	DP056_003
CZE-06			KC536544	*D. pulex*	EPX	49.42	13.78	Blatná	Czech Republic	this paper	DP056_004
CZE-07	KC536303, KC536304	KC535996, KC535997	KC536545	*D. pulex*	EPX	49.42	13.78	Blatná	Czech Republic	this paper	DP056_010
CZE-08	KC536305, KC536306			*D. pulex*	missing	49.42	13.78	Blatná	Czech Republic	this paper	DP056_021
CZE-09	KC536307, KC536308		KC536546	*D. pulicaria*	EPC	50.66	13.94	Chabařovice	Czech Republic	this paper	DP057_003
CZE-10		KC536000, KC536001	KC536546	*D. pulicaria*	EPC	50.66	13.94	Chabařovice	Czech Republic	this paper	DP071_01M
CZE-11			KC536546	*D. pulicaria*	EPC	50.66	13.94	Chabařovice	Czech Republic	this paper	DP071_004
CZE-12	KC536317, KC536318			*D. pulicaria*	missing	50.66	13.94	Chabařovice	Czech Republic	this paper	DP071_05M
CZE-13	KC536319, KC536320			*D. pulicaria*	missing	50.66	13.94	Chabařovice	Czech Republic	this paper	DP071_008
CZE-14	KC536309, KC536310	KC535998, KC535999	KC536547	*D. pulicaria*	EPC	49.44	13.78	Blatná	Czech Republic	this paper	DP058_004

CZE-15			KC536548	*D. pulicaria*	EPC	49.44	13.78	Blatná	Czech Republic	this paper	DP058_005
CZE-16	KC536311, KC536312		KC536549	*D. pulicaria*	EPC	49.44	13.78	Blatná	Czech Republic	this paper	DP058_006
CZE-17	KC536313, KC536314			*D. pulicaria*	missing	49.44	13.78	Blatná	Czech Republic	this paper	DP058_007
CZE-18	KC536315, KC536316		KC536550	*D. pulicaria*	EPC	50.20	13.91	Mšec	Czech Republic	this paper	DP064_002
CZE-19	HQ434704		FJ591100	*D. pulicaria*	EPC	49.41	13.77	Blatna	Czech Republic	Vergilino et al. 2011	Chmelnice
CZE-20	HQ434748		HQ434662	*D. pulicaria*	EPC	49.40	13.81	Blatná	Czech Republic	Vergilino et al. 2011	Mala_kus_5
CZE-21		JN117728, JN117729		*D. pulicaria*	EPC	50.08	14.48		Czech Republic	Crease et al. 2011	EPC01
CZE-22		JN117730		*D. pulicaria*	EPC	50.18	12.88		Czech Republic	Crease et al. 2011	EPC03_CZR
CZE-23		JN117731,JN117732		*D. pulex*	EPX	48.88	14.63		Czech Republic	Crease et al. 2011	EPX_CZR
DEU-01	KC536431, KC536432	KC536065, KC536066	KC536593	*D. pulex*	EPX	49.00	12.15	Regensburg	Gernamy	this paper	DP165_001
DEU-02	KC536433, KC536434	KC536067, KC536068	KC536593	*D. pulex*	EPX	49.00	12.15	Regensburg	Gernamy	this paper	DP165_002
DEU-03			KC536546	*D. pulicaria*	EPC	54.32	10.63	Grosser Binnensee	Germany	this paper	L16
ESP-01	KC536171, KC536172		KC536512	*D. pulicaria*	EPC	37.05	-3.33	Sierra Nevada	Spain	this paper	DP019_001
ESP-02	KC536173, KC536174		KC536513	*D. pulicaria*	EPC	37.05	-3.33	Sierra Nevada	Spain	this paper	DP019_005
ESP-03	KC536175, KC536176		KC536514	*D. pulicaria*	EPC	37.05	-3.33	Sierra Nevada	Spain	this paper	DP019_006
ESP-04	KC536177, KC536178		KC536513	*D. pulicaria*	EPC	37.05	-3.33	Sierra Nevada	Spain	this paper	DP019_007
ESP-05	KC536179, KC536180		KC536514	*D. pulicaria*	EPC	37.05	-3.33	Sierra Nevada	Spain	this paper	DP019_008
ESP-06			KC536513	*D. pulicaria*	EPC	37.05	-3.33	Sierra Nevada	Spain	this paper	DP019_009
ESP-07	KC536181, KC536182		KC536515	*D. pulicaria*	EPC	37.05	-3.34	Sierra Nevada	Spain	this paper	DP020_002
ESP-08	KC536183, KC536184		KC536516	*D. pulicaria*	EPC	37.05	-3.34	Sierra Nevada	Spain	this paper	DP020_005
ESP-09	KC536185, KC536186		KC536516	*D. pulicaria*	EPC	37.05	-3.34	Sierra Nevada	Spain	this paper	DP020_006
ESP-10	KC536187, KC536188		KC536516	*D. pulicaria*	EPC	37.05	-3.34	Sierra Nevada	Spain	this paper	DP020_007
ESP-11	KC536189, KC536190		KC536516	*D. pulicaria*	EPC	37.05	-3.34	Sierra Nevada	Spain	this paper	DP020_008
ESP-12			KC536517	*D. pulicaria*	EPC	37.05	-3.34	Sierra Nevada	Spain	this paper	DP020_009
ESP-13	KC536191, KC536192			*D. pulicaria*	missing	42.66	1.39	Pyrenees	Spain	this paper	DP021_003
ESP-14	KC536193, KC536194		KC536518	*D. pulicaria*	EPC	42.66	1.39	Pyrenees	Spain	this paper	DP021_005
ESP-15	KC536195, KC536196		KC536519	*D. pulicaria*	EPC	42.66	1.39	Pyrenees	Spain	this paper	DP021_006
ESP-16	KC536197, KC536198		KC536519	*D. pulicaria*	EPC	42.66	1.39	Pyrenees	Spain	this paper	DP021_18M
ESP-17	KC536199, KC536200		KC536574	*D. pulicaria*	EPC	42.66	1.39	Pyrenees	Spain	this paper	DP105_001
ESP-18	KC536201, KC536202		KC536519	*D. pulicaria*	EPC	42.65	1.38	Pyrenees	Spain	this paper	DP022_001
ESP-19			KC536520	*D. pulicaria*	EPC	42.65	1.38	Pyrenees	Spain	this paper	DP022_008
ESP-20	KC536203, KC536204	KC535971, KC535972	KC536519	*D. pulicaria*	EPC	42.65	1.38	Pyrenees	Spain	this paper	DP022_010
ESP-21		KC535973, KC535974	KC536521	*D. pulicaria*	EPC	42.65	1.38	Pyrenees	Spain	this paper	DP022_011
ESP-22	KC536205, KC536206		KC536522	*D. pulicaria*	EPC	42.65	1.38	Pyrenees	Spain	this paper	DP022_17M
ESP-23	KC536207, KC536208		KC536523	*D. pulicaria*	EPC	42.64	0.78	Pyrenees	Spain	this paper	DP023_007
ESP-24	KC536209, KC536210		KC536524	*D. pulicaria*	EPC	42.64	0.78	Pyrenees	Spain	this paper	DP023_008
ESP-25	KC536211, KC536212		KC536523	*D. pulicaria*	EPC	42.64	0.78	Pyrenees	Spain	this paper	DP023_009
ESP-26		KC535975, KC535976	KC536523	*D. pulicaria*	EPC	42.64	0.78	Pyrenees	Spain	this paper	DP023_010
ESP-27	KC536213, KC536214	KC535977, KC535978	KC536523	*D. pulicaria*	EPC	42.64	0.78	Pyrenees	Spain	this paper	DP023_11M
ESP-28	KC536215, KC536216	KC535979, KC535980	KC536523	*D. pulicaria*	EPC	42.64	0.78	Pyrenees	Spain	this paper	DP023_17M
ESP-29			KC536575	*D. pulicaria*	EPC	42.64	0.78	Pyrenees	Spain	this paper	DP106_001
ESP-30			KC536523	*D. pulicaria*	EPC	42.64	0.78	Pyrenees	Spain	this paper	DP106_002
ESP-31			KC536542	*D. pulicaria*	EPC	41.00	-3.50	San Rafael de Matallana	Spain	this paper	DP047_001
ESP-32	HQ434761		HQ434652	*D. pulicaria*	EPC	42.63	1.00	Pyrenees	Spain	Vergilino et al. 2011	Ger1a
ESP-33		JN117728		*D. pulicaria*	EPC	42.63	1.00	Pyrenees	Spain	Crease et al. 2011	EPC02_SPA
GBR-01	KC536293, KC536294	KC535994, KC535995	KC536541	*D. pulicaria*	EPC	51.39	-0.40	London	United Kingdom	this paper	DP044_001
GBR-02	KC536295, KC536296		KC536541	*D. pulicaria*	EPC	51.65	-0.02	London	United Kingdom	this paper	DP045_003
GBR-03	KC536385, KC536386	KC536017, KC536018	KC536576	*D. pulicaria*	EPC	56.75	-4.90	Ben Nevis	United Kingdom	this paper	DP118_001
GBR-04			KC536577	*D. pulicaria*	EPC	56.75	-4.90	Ben Nevis	United Kingdom	this paper	DP118_002
GBR-05			KC536576	*D. pulicaria*	EPC	56.75	-4.90	Ben Nevis	United Kingdom	this paper	DP118_003
GBR-06	KC536435, KC536436	KC536071, KC536072	KC536555	*D. pulex*	EPX	52.14	0.35	West Wratting	United Kingdom	this paper	DP167_001
GBR-07	KC536437, KC536438	KC536073, KC536074	KC536555	*D. pulex*	EPX	52.14	0.35	West Wratting	United Kingdom	this paper	DP168_001
GBR-08	KC536439, KC536440	KC536075, KC536076	KC536555	*D. pulex*	EPX	52.10	0.37	Streetly End	United Kingdom	this paper	DP169_001
GRL-01	KC536410, KC536411, KC536412	KC536041, KC536042, KC536043	KC536586	*D. pulicaria*	NAPC	64.15	-51.31	Nuuk	Greenland	this paper	DP132_001
GRL-02			KC536586	*D. pulicaria*	NAPC	64.15	-51.31	Nuuk	Greenland	this paper	DP132_002

GRL-03	KC536413, KC536414, KC536415	KC536044, KC536045	KC536586	*D. pulicaria*	NAPC	64.15	-51.31	Nuuk	Greenland	this paper	DP132_003
GRL-04	KC536416, KC536417, KC536418	KC536046, KC536047	KC536586	*D. pulicaria*	NAPC	64.15	-51.31	Nuuk	Greenland	this paper	DP132_004
GRL-05	KC536494, KC536495	KC536126, KC536127	KC536620	Hybrid *D. pulicaria* X *D. pulex*	NAPC	69.21	-51.10	Jakobshavn	Greenland	this paper	L1
HUN-01			KC536541	*D. pulicaria*	EPC	47.57	21.16	Hortobagy	Hungaria	this paper	DP046_003
ID-01	EU918527, EU918528			*D. pulicaria*	NAPC	42.35	-112.44	Idaho	USA	Omilian et al. 2008	DAN15_ID
ID-02		JN117740, JN117735		*D. pulicaria*	NAPX	42.35	-112.44	Idaho	USA	Crease et al. 2011	C08_ID
IL-01	KC536454, KC536455		KC536599	*D. pulex*	NAPX	40.13	-88.22	Busey woods, Illinois	USA	this paper	DX011_001
IL-02	HQ434752		HQ434667	*D. pulicaria*	NAPX	40.12	-87.70	Vermillion Country, Illinois	USA	Vergilino et al. 2011	Puli9
IL-03	HQ434753		HQ434669	*D. pulicaria*	NAPX	40.12	-87.70	Vermillion Country, Illinois	USA	Vergilino et al. 2011	Puli15
IL-04	EU918457, EU918458			*D.pulex*	NAPX	40.20	-89.00	Illinois	USA	Omilian et al. 2008	West2_IL
IL-05	EU918459, EU918460			*D.pulex*	NAPX	40.20	-89.00	Illinois	USA	Omilian et al. 2008	West5_IL
IL-06		JN117733		*D. pulicaria*	NAPX	40.14	-87.74	Illinois	USA	Crease et al. 2011	C07_IL
IL-07		JN117733		*D. pulicaria*	NAPX	40.13	-87.73	Illinois	USA	Crease et al. 2011	C09_IL
IL-08		JN117733		*D. pulicaria*	NAPX	40.15	-87.74	Illinois	USA	Crease et al. 2011	C10_IL
IL-09		JN117762, JN117770		*D.pulex*	NAPX	40.12	-88.20	Illinois	USA	Crease et al. 2011	X02_IL
IL-10		JN117772, JN117773		*D.pulex*	NAPX	40.06	-87.92	Illinois	USA	Crease et al. 2011	X04_IL
IL-11		JN117776, JN117777		*D.pulex*	NAPX	40.14	-87.74	Illinois	USA	Crease et al. 2011	X08_IL
IL-12		JN117762, JN117800		*D.pulex*	NAPX	41.69	-89.27	Illinois	USA	Crease et al. 2011	X35_IL
IN-01	KC536456, KC536457	KC536085, KC536086	KC536600	*D. pulicaria*	NAPC	39.90	-85.43	Indiana	USA	this paper	DX012_001
IN-02	KC536458, KC536459	KC536087, KC536088	KC536601	*D. pulicaria*	NAPC	39.90	-85.43	Indiana	USA	this paper	DX013_001
IN-03	HQ434758		FJ591104	*D.pulicaria*	NAPC	40.00	-86.00	Indiana	USA	Vergilino et al. 2011	W4_95
IN-04	EU918461, EU918462			*D. pulex*	NAPX	40.00	-86.00	Indiana	USA	Omilian et al. 2008	SAL5_IN
IN-05	EU918463, EU918464			*D. pulex*	NAPX	40.00	-86.00	Indiana	USA	Omilian et al. 2008	SAL7_IN
IN-06	EU918465, EU918466			*D. pulex*	NAPX	40.00	-86.00	Indiana	USA	Omilian et al. 2008	PA27_IN
IN-07	EU918467, EU918468			*D. pulex*	NAPX	40.00	-86.00	Indiana	USA	Omilian et al. 2008	PA35_IN
IN-08		JN117759, JN117760		Hybrid *D. pulicaria* X *D. pulex*	NAPX	39.12	-87.31	Indiana	USA	Crease et al. 2011	CX07_IN
IN-09		JN117779		*D.pulex*	NAPX	39.90	-84.93	Indiana	USA	Crease et al. 2011	X10_IN
IN-10		JN117780, JN117781		*D.pulex*	NAPX	40.22	-87.33	Indiana	USA	Crease et al. 2011	X11_IN
ISL-01	KC536408, KC536409	KC536038, KC536039, KC536040	KC536585	*D. pulicaria*	EPC	64.14	-21.94	Reykjavik	Iceland	this paper	DP131_001
ISL-02			KC536585	*D. pulicaria*	EPC	64.14	-21.94	Reykjavik	Iceland	this paper	DP131_002
ISL-03			KC536585	*D. pulicaria*	EPC	64.14	-21.94	Reykjavik	Iceland	this paper	DP131_003
ISL-04	KC536496, KC536497, KC536498	KC536128, KC536129	KC536621	*D. pulicaria*	NAPC	64.13	-21.94	Reykjavik	Iceland	this paper	L2
ISL-05		JN117734, JN117751		Hybrid *D. pulicaria* X *D. pulex*	NAPC	64.09	-21.58	Reykjavik	Iceland	Crease et al. 2011	CX01_ICE
ITA-01	KC536217, KC536218	KC535981, KC535982	KC536525	*D. pulicaria*	EPC	46.48	11.66	Dolomites	Italy	this paper	DP024_002
ITA-02	KC536219, KC536220		KC536525	*D. pulicaria*	EPC	46.48	11.66	Dolomites	Italy	this paper	DP024_005
ITA-03	KC536221, KC536222		KC536525	*D. pulicaria*	EPC	46.48	11.66	Dolomites	Italy	this paper	DP024_006
ITA-04	KC536223, KC536224		KC536525	*D. pulicaria*	EPC	46.48	11.66	Dolomites	Italy	this paper	DP024_007
ITA-05	KC536225, KC536226		KC536525	*D. pulicaria*	EPC	46.48	11.66	Dolomites	Italy	this paper	DP024_008
ITA-06			KC536525	*D. pulicaria*	EPC	46.48	11.66	Dolomites	Italy	this paper	DP024_009
ITA-06	KC536363, KC536364		KC536565	Hybrid *D. pulicaria* X *D. pulex*	NAPX	45.07	7.39	Avigliana	Italy	this paper	DP085_001
ITA-07	KC536365, KC536366	KC536010, KC536011	KC536565	Hybrid *D. pulicaria* X *D. pulex*	NAPX	45.07	7.39	Avigliana	Italy	this paper	DP085_003
ITA-08			KC536565	Hybrid *D. pulicaria* X *D. pulex*	NAPX	45.07	7.39	Avigliana	Italy	this paper	DP085_004
ITA-09			KC536565	Hybrid *D. pulicaria* X *D. pulex*	NAPX	45.07	7.39	Avigliana	Italy	this paper	DP085_005
ITA-10			KC536563	*D. pulicaria*	EPC	45.07	7.39	Avigliana	Italy	this paper	DP085_010
ITA-11			KC536565	Hybrid *D. pulicaria* X *D. pulex*	NAPX	45.07	7.39	Avigliana	Italy	this paper	DP085_011
ITA-12			KC536565	Hybrid *D. pulicaria* X *D. pulex*	NAPX	45.07	7.39	Avigliana	Italy	this paper	DP085_012
ITA-13			KC536565	Hybrid *D. pulicaria* X *D. pulex*	NAPX	45.07	7.39	Avigliana	Italy	this paper	DP085_013
ITA-14	KC536367, KC536368	KC536570		Hybrid *D. pulicaria* X *D. pulex*	NAPX	45.07	7.39	Avigliana	Italy	this paper	DP100_003
ITA-15	KC536369, KC536370		KC536565	Hybrid *D. pulicaria* X *D. pulex*	NAPX	45.07	7.39	Avigliana	Italy	this paper	DP101_001
ITA-16			KC536565	Hybrid *D. pulicaria* X *D. pulex*	NAPX	45.07	7.39	Avigliana	Italy	this paper	DP101_002
ITA-17			KC536565	Hybrid *D. pulicaria* X *D. pulex*	NAPX	45.07	7.39	Avigliana	Italy	this paper	DP101_003
ITA-18	KC536371, KC536372	KC536069, KC536070	KC536594	Hybrid *D. pulicaria* X *D. pulex*	NAPX	45.07	7.39	Avigliana	Italy	this paper	DP166_001
ITA-19	KC536373, KC536374		KC536565	Hybrid *D. pulicaria* X *D. pulex*	NAPX	45.07	7.39	Avigliana	Italy	this paper	DP166_002
ITA-20				Hybrid *D. pulicaria* X *D. pulex*	NAPX	40.56	9.32	Sardinia	Italy	this paper	DP102_002
ITA-21			KC536565	Hybrid *D. pulicaria* X *D. pulex*	NAPX	40.56	9.32	Sardinia	Italy	this paper	DP102_003

ITA-22			KC536565	Hybrid *D. pulicaria* X *D. pulex*	NAPX	40.56	9.32	Sardinia	Italy	this paper	DP102_004
KGZ-01			KC536566	*D. pulicaria*	NAPC	42.36	73.80	Kara Balta	Kyrgyzstan	this paper	DP087_001
KGZ-02	KC536375, KC536376	KC536012	KC536567	*D. pulicaria*	NAPC	42.36	73.80	Kara Balta	Kyrgyzstan	this paper	DP087_002
KGZ-03	KC536377, KC536378	KC536013, KC536014	KC536566	*D. pulicaria*	NAPC	42.36	73.80	Kara Balta	Kyrgyzstan	this paper	DP087_003
KGZ-04			KC536568	*D. pulicaria*	NAPC	42.36	73.80	Kara Balta	Kyrgyzstan	this paper	DP087_004
KGZ-05	KC536379, KC536380		KC536566	*D. pulicaria*	NAPC	42.36	73.80	Kara Balta	Kyrgyzstan	this paper	DP087_005
KGZ-06			KC536566	*D. pulicaria*	NAPC	42.36	73.80	Kara Balta	Kyrgyzstan	this paper	DP087_006
KGZ-07			KC536566	*D. pulicaria*	NAPC	42.36	73.80	Kara Balta	Kyrgyzstan	this paper	DP087_010
KGZ-08			KC536566	*D. pulicaria*	NAPC	42.36	73.80	Kara Balta	Kyrgyzstan	this paper	DP087_011
LTU-01	KC536339, KC536340		KC536560	*D. pulex*	EPX	54.75	25.29	Vilnius	Lithuania	this paper	DP081_001
LTU-02			KC536561	*D. pulex*	EPX	54.75	25.29	Vilnius	Lithuania	this paper	DP081_002
LTU-03				*D. pulex*	EPX	54.75	25.29	Vilnius	Lithuania	this paper	DP081_003
LTU-04	KC536341, KC536342	KC536008, KC536009	KC536562	*D. pulex*	EPX	54.69	25.24	Vilnius	Lithuania	this paper	DP082_002
LTU-05			KC536561	*D. pulex*	EPX	54.69	25.24	Vilnius	Lithuania	this paper	DP082_003
LTU-06			KC536561	*D. pulex*	EPX	54.69	25.24	Vilnius	Lithuania	this paper	DP082_004
MB-01	KC536464, KC536465	KC536093, KC536094	KC536604	*D. pulex*	NAPX	58.77	-94.17	Churchill, Manitoba	Canada	this paper	DX016_001
MB-02	KC536468, KC536469, KC536470	KC536096, KC536097, KC536098	KC536605	*D. tenebrosa*	TEN	58.77	-94.17	Churchill, Manitoba	Canada	this paper	DX018_001
MB-03	KC536483, KC536484, KC536485	KC536107, KC536108, KC536109	KC536610	*D. middendorffiana*	MID	58.78	-94.19	Churchill, Manitoba	Canada	this paper	DX022_001
MB-04			KC536610	*D. middendorffiana*	MID	58.78	-94.19	Churchill, Manitoba	Canada	this paper	DX022_002
MB-05			KC536610	*D. middendorffiana*	MID	58.78	-94.19	Churchill, Manitoba	Canada	this paper	DX022_003
MB-06	KC536486, KC536487	KC536110, KC536111	KC536611	*D. pulicaria*	NAPC	52.12	-97.25	Winnipeg, Manitoba	Canada	this paper	DX023_001
MB-07			KC536611	*D. pulicaria*	NAPC	52.12	-97.25	Winnipeg, Manitoba	Canada	this paper	DX023_002
MB-08		KC536112, KC536113	KC536612	*D. tenebrosa*	TEN	58.74	-93.96	Churchill, Manitoba	Canada	this paper	DX024_001
MB-09	KC536488, KC536489		KC536612	*D. tenebrosa*	TEN	58.74	-93.96	Churchill, Manitoba	Canada	this paper	DX024_002
MB-10	KC536490, KC536491	KC536114, KC536115	KC536613	*D. tenebrosa*	TEN	58.77	-93.97	Churchill, Manitoba	Canada	this paper	DX025_001
MB-11			KC536613	*D. tenebrosa*	TEN	58.77	-93.97	Churchill, Manitoba	Canada	this paper	DX025_002
MB-12	KC536492, KC536493	KC536116, KC536117, KC536118	KC536610	*D. middendorffiana*	MID	58.78	-94.19	Churchill, Manitoba	Canada	this paper	DX026_001
MB-13			KC536610	*D. middendorffiana*	MID	58.78	-94.19	Churchill, Manitoba	Canada	this paper	DX026_002
MB-14			KC536610	*D. middendorffiana*	MID	58.78	-94.19	Churchill, Manitoba	Canada	this paper	DX026_003
MB-15			KC536614	*D. pulicaria*	NAPC	58.77	-94.17	Churchill, Manitoba	Canada	this paper	A20_001
MB-16	KC536132, KC536133, KC536134		KC536614	*D. pulicaria*	NAPC	58.77	-94.17	Churchill, Manitoba	Canada	this paper	A20_003
MB-17	KC536449, KC536450		KC536619	*D. tenebrosa*	TEN	58.77	-94.17	Churchill, Manitoba	Canada	this paper	T59
MB-18	HQ434691		FJ591107	*D. pulex*	NAPX	58.73	-94.09	Churchill, Manitoba	Canada	Vergilino et al. 2011	A17_200
MB-19	HQ434698		FJ591106	*Hybrid D. pulicaria X D. pulex*	NAPC	58.73	-94.09	Churchill, Manitoba	Canada	Vergilino et al. 2011	B141_202
MB-20	HQ434709, HQ434710, HQ434711		FJ591116	*D. tenebrosa*	TEN	58.77	-94.18	Churchill, Manitoba	Canada	Vergilino et al. 2011	Clone_B
MB-21	HQ434712		FJ591119	*D. tenebrosa*	TEN	58.77	-94.18	Churchill, Manitoba	Canada	Vergilino et al. 2011	Clone_D
MB-22	HQ434713		FJ591118	*D. tenebrosa*	TEN	58.77	-94.18	Churchill, Manitoba	Canada	Vergilino et al. 2011	Clone_E
MB-23	HQ434760		HQ434689	*D.pulicaria*	NAPC	49.90	-97.15	Winnipeg. Manitoba	Canada	Vergilino et al. 2011	winni1
MB-24		JN117740, JN117735		*D.pulicaria*	NAPX	49.16	-96.30	Manitoba	Canada	Crease et al. 2011	C15_MB
MB-25		JN117743, JN117740		*D.pulicaria*	NAPX	50.04	-100.32	Manitoba	Canada	Crease et al. 2011	C16_MB
MB-26		JN117745, JN117746, JN117740		*D. middendorffiana*	MID	58.46	-93.51	Manitoba	Canada	Crease et al. 2011	MID01_MB
MB-27		JN117740, JN117765		Hybrid *D. pulicaria* X *D. pulex*	NAPX	49.64	-98.24	Manitoba	Canada	Crease et al. 2011	CX15_MB
MB-28		JN117766, JN117767		Hybrid *D. pulicaria* X *D. pulex*	NAPX	49.64	-98.24	Manitoba	Canada	Crease et al. 2011	CX16_MB
ME-01	EU918529, EU918530			*D. pulicaria*	NAPC	44.84	-69.28	Maine	USA	Omilian et al. 2008	MOOSE_ME
ME-02	EU918445, EU918446			*D. pulex*	NAPX	44.84	-69.28	Maine	USA	Omilian et al. 2008	PHM1_ME
ME-03	EU918453, EU918454			*D.pulex*	NAPX	44.84	-69.28	Maine	USA	Omilian et al. 2008	TRO3_ME
ME-04		JN117734		*D. pulicaria*	NAPC	44.84	-69.28	Maine	USA	Crease et al. 2011	C02_ME
ME-05		JN117740, JN117754		Hybrid *D. pulicaria* X *D. pulex*	NAPX	42.99	-70.61	Maine	USA	Crease et al. 2011	CX04_ME
ME-06		JN117740, JN117756		Hybrid *D. pulicaria* X *D. pulex*	NAPX	44.86	-69.80	Maine	USA	Crease et al. 2011	CX10_ME
ME-07		JN117761		*D.pulex*	NAPX	46.50	-68.22	Maine	USA	Crease et al. 2011	X26_ME
ME-08		JN117761, JN117783		*D.pulex*	NAPX	44.63	-69.23	Maine	USA	Crease et al. 2011	X29_ME
MI-01	HQ434732		HQ434655	*Hybrid D. pulicaria X D. pulex*	NAPX	44.00	-85.00	Michigan	USA	Vergilino et al. 2011	IWR10
MI-02	EU918531, EU918532			*D. pulicaria*	NAPC	44.00	-85.00	Michigan	USA	Omilian et al. 2008	BAKER_MI
MI-03	EU918431, EU918432			*D. pulex*	NAPX	44.00	-85.00	Michigan	USA	Omilian et al. 2008	CR1_MI
MI-04	EU918533, EU918534			*D. pulicaria*	NAPC	42.65	-85.50	Michigan	USA	Omilian et al. 2008	LL_MI

MI-05	EU918439, EU918440			*D. pulex*	NAPX	42.65	-85.50	Michigan	USA	Omilian et al. 2008	LYT1_MI
MI-06	EU918469, EU918470			*D. pulex*	NAPX	42.65	-85.50	Michigan	USA	Omilian et al. 2008	MAR6_MI
MI-07	EU918471, EU918472			*D. pulex*	NAPX	42.65	-85.50	Michigan	USA	Omilian et al. 2008	MAR8_MI
MI-08	EU918473, EU918474			*D. pulex*	NAPX	42.65	-85.50	Michigan	USA	Omilian et al. 2008	NDB3_MI
MI-09	EU918475, EU918476			*D. pulex*	NAPX	42.65	-85.50	Michigan	USA	Omilian et al. 2008	NDB4_MI
MI-10		JN117735		*D. pulicaria*	NAPC	42.65	-85.50	Michigan	USA	Crease et al. 2011	C03_MI
MI-11		JN117740, JN117753		Hybrid *D. pulicaria* X *D. pulex*	NAPX	42.21	-83.70	Michigan	USA	Crease et al. 2011	CX03_MI
MI-12		JN117755, JN117754		Hybrid *D. pulicaria* X *D. pulex*	NAPX	42.17	-83.72	Michigan	USA	Crease et al. 2011	CX11_MI
MI-13		JN117768, JN117769		*D.pulex*	NAPX	42.19	-83.58	Michigan	USA	Crease et al. 2011	X01_MI
MI-14		JN117782, JN117768		*D.pulex*	NAPX	42.75	-85.35	Michigan	USA	Crease et al. 2011	X12_MI
MI-15		JN117783, JN117778		*D.pulex*	NAPX	42.20	-83.72	Michigan	USA	Crease et al. 2011	X13_MI
MI-16		JN117768, JN117778		*D.pulex*	NAPX	42.33	-83.66	Michigan	USA	Crease et al. 2011	X16_MI
MI-17		JN117783, JN117761		*D.pulex*	NAPX	46.06	-86.79	Michigan	USA	Crease et al. 2011	X19_MI
MI-18		JN117761		*D.pulex*	NAPX	46.01	-86.66	Michigan	USA	Crease et al. 2011	X24_MI
MN-01	EU918441, EU918442			*D. pulex*	NAPX	46.00	-94.00	Minnesota	USA	Omilian et al. 2008	NOS1_MN
MN-02	EU918477, EU918478			*D. pulex*	NAPX	46.00	-94.00	Minnesota	USA	Omilian et al. 2008	MPP1_MN
MN-03	EU918479, EU918480			*D. pulex*	NAPX	46.00	-94.00	Minnesota	USA	Omilian et al. 2008	MPP3_MN
MN-04	EU918481, EU918482			*D. pulex*	NAPX	46.00	-94.00	Minnesota	USA	Omilian et al. 2008	EB1_MN
MN-05	EU918483, EU918484			*D. pulex*	NAPX	46.00	-94.00	Minnesota	USA	Omilian et al. 2008	EB4_MN
MN-06		JN117761, JN117775		*D.pulex*	NAPX	44.97	-93.32	Minnesota	USA	Crease et al. 2011	X06_MN
MN-07		JN117791, JN117792		*D.pulex*	NAPX	47.29	-92.49	Minnesota	USA	Crease et al. 2011	X25_MN
MNE-01	KC536227, KC536228	KC535983, KC535984	KC536526	*D. pulicaria*	EPC	43.12	19.04	Durmintor	Monte Negro	this paper	DP035_001
MNE-02	KC536229, KC536230		KC536527	*D. pulicaria*	EPC	43.12	19.04	Durmintor	Monte Negro	this paper	DP035_005
MNE-03	KC536231, KC536232		KC536527	*D. pulicaria*	EPC	43.12	19.04	Durmintor	Monte Negro	this paper	DP035_006
MNE-04	KC536233, KC536234		KC536528	*D. pulicaria*	EPC	43.12	19.04	Durmintor	Monte Negro	this paper	DP035_007
MNE-05	KC536235, KC536236		KC536527	*D. pulicaria*	EPC	43.12	19.04	Durmintor	Monte Negro	this paper	DP035_008
MNE-06			KC536527	*D. pulicaria*	EPC	43.12	19.04	Durmintor	Monte Negro	this paper	DP035_009
NB-01		JN117755, JN117756		Hybrid *D. pulicaria* X *D. pulex*	NAPX	47.00	-67.47	New Brunswick	Canada	Crease et al. 2011	CX05_NB
NB-02		JN117757, JN117758		Hybrid *D. pulicaria* X *D. pulex*	NAPX	47.00	-67.47	New Brunswick	Canada	Crease et al. 2011	CX06_NB
NB-03		JN117735, JN117762		Hybrid *D. pulicaria* X *D. pulex*	NAPX	47.62	-65.67	New Brunswick	Canada	Crease et al. 2011	CX12_NB
NOR-01	KC536287, KC536288	KC535992, KC535993	KC536539	*D. pulicaria*	EPC	60.28	7.53	Hardangervidda	Norway	this paper	DP042_001
NOR-02	KC536289, KC536290		KC536539	*D. pulicaria*	EPC	60.28	7.53	Hardangervidda	Norway	this paper	DP042_002
NOR-03	KC536291, KC536292		KC536540	*D. pulicaria*	EPC	60.28	7.53	Hardangervidda	Norway	this paper	DP042_003
NU-01	KC536135, KC536136			*D. pulicaria*	missing	72.90	-79.85	Bylot Island, Nunavut	Canada	this paper	B_003
NU-02	KC536137, KC536138	KC536121, KC536122, KC536123	KC536617	*D. pulicaria*	NAPC	72.90	-79.85	Bylot Island, Nunavut	Canada	this paper	B_004
NU-03			KC536617	*D. pulicaria*	NAPC	72.90	-79.85	Bylot Island, Nunavut	Canada	this paper	B_005
NU-04			KC536617	*D. pulicaria*	NAPC	72.90	-79.85	Bylot Island, Nunavut	Canada	this paper	B_006
NU-05			KC536617	*D. pulicaria*	NAPC	72.90	-79.85	Bylot Island, Nunavut	Canada	this paper	B_007
NU-06			KC536617	*D. pulicaria*	NAPC	72.90	-79.85	Bylot Island, Nunavut	Canada	this paper	B_008
NU-07		JN117745, JN117747		*D. middendorffiana*	MID	72.68	-77.94	Nunavut	Canada	Crease et al. 2011	MID02
NU-08		JN117914, JN117915		*D. tenebrosa*	TEN	70.29	-127.50	Nunavut	Canada	Crease et al. 2011	TEN_NUN
NY-01	EU918429, EU918430			*D. pulex*	NAPX	44.00	-74.00	New York	USA	Omilian et al. 2008	AST1_NY
NY-02		JN117787, JN117778		*D.pulex*	NAPX	42.98	-78.77	New York	USA	Crease et al. 2011	X18_NY
NY-03		JN117778		*D.pulex*	NAPX	42.47	-76.37	New York	USA	Crease et al. 2011	X27_NY
NT-01		JN117740, JN117763		Hybrid *D. pulicaria* X *D. pulex*	NAPX	69.43	-133.02	Northwest Territories	Canada	Crease et al. 2011	CX13_NT
OH-01	EU918455, EU918456			*D.pulex*	NAPX	40.20	-82.60	Ohio	USA	Omilian et al. 2008	WCH1_OH
OH-02		JN117762, JN117795		*D.pulex*	NAPX	41.34	-81.16	Ohio	USA	Crease et al. 2011	X30_OH
ON-01	KC536451, KC536452, KC536453		KC536565	Hybrid *D. pulicaria* X *D. pulex*	NAPX	42.22	-83.03	Disputed Road, Ontario	Canada	this paper	DX010_001
ON-02	KC536460, KC536461	KC536089, KC536090	KC536602	*D. pulex*	NAPX	43.73	-80.95	Listowel pond, Ontario	Canada	this paper	DX014_001
ON-03	HQ434719		HQ434640	*D. pulex*	NAPX	42.22	-83.03	Disputed Road, Ontario	Canada	Vergilino et al. 2011	Disp13
ON-04	HQ434700		HQ434632	*D. pulex*	NAPX	52.20	-87.30	Windsor, Ontario	Canada	Vergilino et al. 2011	Can2_1
ON-05	HQ434729		HQ434649	Hybrid *D. pulicaria* X *D. pulex*	NAPX	52.20	-87.30	Windsor, Ontario	Canada	Vergilino et al. 2011	Fence1
ON-06	EU918437, EU918438			*D. pulex*	NAPX	45.10	-77.97	Ontario	Canada	Omilian et al. 2008	Hughes2
ON-07	EU918541, EU918542			*D. pulicaria*	NAPC	45.10	-77.97	Ontario	Canada	Omilian et al. 2008	MINER_ONT
ON-08	EU918443, EU918444			*D. pulex*	NAPX	45.10	-77.97	Ontario	Canada	Omilian et al. 2008	PETE1_ON

ON-09	EU918447, EU918448			*D. pulex*	NAPX	45.10	-77.97	Ontario	Canada	Omilian et al. 2008	RONIII_ONT
ON-10		JN117733		*D. pulicaria*	NAPC	44.88	-78.75	Ontario	Canada	Crease et al. 2011	C01_ON
ON-11		JN117740, JN117761		Hybrid *D. pulicaria* X *D. pulex*	NAPX	42.12	-82.98	Ontario	Canada	Crease et al. 2011	CX08_ON
ON-12		JN117740, JN117761		Hybrid *D. pulicaria* X *D. pulex*	NAPX	42.16	-83.02	Ontario	Canada	Crease et al. 2011	CX09_ON
ON-13		JN117735, JN117764		Hybrid *D. pulicaria* X *D. pulex*	NAPX	49.82	-93.23	Ontario	Canada	Crease et al. 2011	CX14_ON
ON-14		JN117774		*D.pulex*	NAPX	42.17	-83.03	Ontario	Canada	Crease et al. 2011	X05_ON
ON-15		JN117761		*D.pulex*	NAPX	42.25	-83.02	Ontario	Canada	Crease et al. 2011	X07_ON
ON-16		JN117778		*D.pulex*	NAPX	42.67	-80.40	Ontario	Canada	Crease et al. 2011	X09_ON
ON-17		JN117783		*D.pulex*	NAPX	43.54	-80.21	Ontario	Canada	Crease et al. 2011	X17_ON
ON-18		JN117793, JN117794		*D.pulex*	NAPX	42.33	-81.84	Ontario	Canada	Crease et al. 2011	X28_ON
ON-19		JN117771		*D.pulex*	NAPX	49.82	-93.23	Ontario	Canada	Crease et al. 2011	X31_ON
OR-01	KC536441, KC536442	KC536077, KC536078	KC536595	*D. arenata*	ARE	44.00	-123.00	Oregon	USA	this paper	DP170_001
OR-02	KC536443, KC536444		KC536596	*D. arenata*	ARE	44.00	-123.00	Oregon	USA	this paper	DP171_001
OR-03	KC536445, KC536446	KC536079, KC536080	KC536597	*D. melanica*	MEL	44.00	-123.00	Oregon	USA	this paper	DP172_001
OR-04	KC536447, KC536448	KC536081, KC536082	KC536598	*D. melanica*	MEL	44.00	-123.00	Oregon	USA	this paper	DP173_001
OR-05	EU918485, EU918486			*D. arenata*	ARE	44.00	-123.00	Oregon	USA	Omilian et al. 2008	Amaz1_OR
OR-06	EU918487, EU918488			*D. arenata*	ARE	44.00	-123.00	Oregon	USA	Omilian et al. 2008	Amaz2_OR
OR-07	EU918489, EU918490			*D. arenata*	ARE	44.00	-123.00	Oregon	USA	Omilian et al. 2008	CC3_OR
OR-08	EU918491, EU918492			*D. arenata*	ARE	44.00	-123.00	Oregon	USA	Omilian et al. 2008	CC6_OR
OR-09	EU918493, EU918494			*D. arenata*	ARE	44.00	-123.00	Oregon	USA	Omilian et al. 2008	CC7_OR
OR-10	EU918515, EU918516			*D. pulex*	NAPX	44.00	-123.00	Oregon	USA	Omilian et al. 2008	GI2_OR
OR-11	EU918517, EU918518			*D. pulex*	NAPX	44.00	-123.00	Oregon	USA	Omilian et al. 2008	GI3_OR
OR-12	EU918519, EU918520			*D. pulex*	NAPX	44.00	-123.00	Oregon	USA	Omilian et al. 2008	GI8_OR
OR-13	EU918521, EU918522			*D. pulex*	NAPX	44.00	-123.00	Oregon	USA	Omilian et al. 2008	GI9_OR
OR-14	EU918523, EU918524			*D. pulex*	NAPX	44.00	-123.00	Oregon	USA	Omilian et al. 2008	GI11_OR
OR-15	EU918525, EU918526			*D. pulex*	NAPX	44.00	-123.00	Oregon	USA	Omilian et al. 2008	GI13_OR
OR-16	EU918503, EU918504			*D. arenata*	ARE	44.00	-123.00	Oregon	USA	Omilian et al. 2008	LOG13_OR
OR-17	EU918505, EU918506			*D. arenata*	ARE	44.00	-123.00	Oregon	USA	Omilian et al. 2008	LOG18_OR
OR-18	EU918507, EU918508			*D. arenata*	ARE	44.00	-123.00	Oregon	USA	Omilian et al. 2008	LOG29_OR
OR-19	EU918509, EU918510			*D. arenata*	ARE	44.00	-123.00	Oregon	USA	Omilian et al. 2008	LOG50_OR
OR-20	EU918513, EU918514			*Hybrid D. pulicaria X D. pulex*	NAPX	44.00	-123.00	Oregon	USA	Omilian et al. 2008	LOG52_OR
OR-21	EU918511, EU918512			*D. arenata*	ARE	44.00	-123.00	Oregon	USA	Omilian et al. 2008	LOG69_OR
OR-22	EU918535, EU918536			*D. pulicaria*	NAPC	44.00	-123.00	Oregon	USA	Omilian et al. 2008	LOSTCR_OR
OR-23	EU918495, EU918496			*D. arenata*	ARE	44.00	-123.00	Oregon	USA	Omilian et al. 2008	OP1_OR
OR-24	EU918497, EU918498			*D. arenata*	ARE	44.00	-123.00	Oregon	USA	Omilian et al. 2008	OP10_OR
OR-25	EU918499, EU918500			*D. arenata*	ARE	44.00	-123.00	Oregon	USA	Omilian et al. 2008	OP11_OR
OR-26	EU918501, EU918502			*D. arenata*	ARE	44.00	-123.00	Oregon	USA	Omilian et al. 2008	OP103_OR
OR-27		JN117725		*D. arenata*	ARE	43.94	-123.05	Oregon	USA	Crease et al. 2011	ARE01_OR
OR-28		JN117726		*D. arenata*	ARE	43.83	-124.12	Oregon	USA	Crease et al. 2011	ARE02_OR
OR-29		JN117727		*D. arenata*	ARE	44.03	-123.15	Oregon	USA	Crease et al. 2011	ARE03_OR
OR-30		JN117744		*D. melanica*	MEL	43.97	-124.11	Oregon	USA	Crease et al. 2011	MEL_OR
OR-31		JN117789		*D. pulex*	NAPX	45.12	-123.03	Oregon	USA	Crease et al. 2011	X21_OR
OR-32	HQ434702, HQ434703		HQ434635	*D. arenata*	ARE	43.92	-123.02	Creswell Court, Oregon	USA	Vergilino et al. 2011	CC3
PA-01	EU918537, EU918538			*D. pulicaria*	NAPC	41.13	-75.58	Pensylvania	USA	Omilian et al. 2008	DUTCH_PA
PA-02		JN117735		*D. pulicaria*	NAPC	41.13	-75.58	Pensylvania	USA	Crease et al. 2011	C04_PA
PER-01		JN117749, JN117750		South American *D. pulicaria*	SAPC	-15.70	-69.50	Titicaca Lake	Peru	Crease et al. 2011	SAPC02
POL-01	KC536155, KC536156		KC536505	*D. pulicaria*	EPC	49.19	20.08	High Tatra Mts.	Poland	this paper	DP003_003
POL-02	KC536157, KC536158		KC536505	*D. pulicaria*	EPC	49.19	20.08	High Tatra Mts.	Poland	this paper	DP003_004
POL-03			KC536505	*D. pulicaria*	EPC	49.19	20.08	High Tatra Mts.	Poland	this paper	DP003_006
POL-04	KC536159, KC536160			*D. pulicaria*	missing	49.19	20.08	High Tatra Mts.	Poland	this paper	DP003_07M
POL-05			KC536506	*D. pulicaria*	EPC	49.19	20.08	High Tatra Mts.	Poland	this paper	DP003_009
POL-06			KC536504	*D. pulicaria*	EPC	49.19	20.08	High Tatra Mts.	Poland	this paper	DP004_02M
POL-07			KC536505	*D. pulicaria*	EPC	49.19	20.08	High Tatra Mts.	Poland	this paper	DP108_001
POL-08			KC536504	*D. pulicaria*	EPC	49.19	20.08	High Tatra Mts.	Poland	this paper	DP108_006
POL-09	KC536381, KC536382	KC536015, KC536016	KC536569	*D. pulex*	EPX	53.80	21.98	Masuria	Poland	this paper	DP096_001

POL-10			KC536569	*D. pulex*	EPX	53.80	21.98	Masuria	Poland	this paper	DP096_002
POL-11	KC536383, KC536384		KC536546	*D. pulicaria*	EPC	53.75	21.78	Masuria	Poland	this paper	DP097_003
POL-12	KC536419, KC536420	KC536048, KC536049	KC536587	*D. pulex*	EPX	50.76	15.70	Giant Mts.	Poland	this paper	DP133_001
POL-13			KC536587	*D. pulex*	EPX	50.76	15.70	Giant Mts.	Poland	this paper	DP133_002
POL-14			KC536587	*D. pulex*	EPX	50.76	15.70	Giant Mts.	Poland	this paper	DP133_003
QC-01	KC536462, KC536463	KC536091, KC536092	KC536603	*D. pulex*	NAPX	48.13	-68.63	Res. Duchesnier, Quebec	Canada	this paper	DX015_001
QC-02	KC536466, KC536467	KC536095	KC536604	Hybrid *D. pulicaria* X *D. pulex*	NAPX	46.76	-71.38	Quebec city, Quebec	Canada	this paper	DX017_001
QC-03	KC536481, KC536482	KC536105, KC536106	KC536609	*D. pulicaria*	NAPC	55.28	-77.75	Kuujjuarapik, Quebec	Canada	this paper	DX021_001
QC-04			KC536609	*D. pulicaria*	NAPC	55.28	-77.75	Kuujjuarapik, Quebec	Canada	this paper	DX021_002
QC-05	KC536141, KC536142	KC536124, KC536125	KC536618	*D. pulicaria*	NAPC	55.28	-77.75	Kuujjuarapik, Quebec	Canada	this paper	Clon2_001
QC-06			KC536618	*D. pulicaria*	NAPC	55.28	-77.75	Kuujjuarapik, Quebec	Canada	this paper	Clon2_002
QC-07			KC536618	*D. pulicaria*	NAPC	55.28	-77.75	Kuujjuarapik, Quebec	Canada	this paper	Clon2_003
QC-08			KC536615	*D. pulicaria*	NAPC	55.28	-77.75	Kuujjuarapik, Quebec	Canada	this paper	n33
QC-09	KC536139, KC536140	KC536119, KC536120	KC536615	*D. pulicaria*	NAPC	55.28	-77.75	Kuujjuarapik, Quebec	Canada	this paper	BGRJ_001
QC-10			KC536616	*D. pulicaria*	NAPC	55.28	-77.75	Kuujjuarapik, Quebec	Canada	this paper	BGRJ_002
QC-11			KC536616	*D. pulicaria*	NAPC	55.28	-77.75	Kuujjuarapik, Quebec	Canada	this paper	BGRJ_003
QC-12	KC536499, KC536500	KC536130, KC536131	KC536622	Hybrid *D. pulicaria* X *D. pulex*	NAPX	55.28	-77.75	Kuujjuarapik, Quebec	Canada	this paper	n37_Dten2
QC-13			KC536617	*D. pulicaria*	NAPC	55.28	-77.75	Kuujjuarapik, Quebec	Canada	this paper	n38_Dten4
QC-14	KC536501, KC536502		KC536623	*D. pulicaria*	NAPC	55.28	-77.75	Kuujjuarapik, Quebec	Canada	this paper	n41_Dten6
QC-15	HQ434731		HQ434651	Hybrid *D. pulicaria* X *D. pulex*	NAPX	46.76	-71.38	Ste-Foy, Quebec	Canada	Vergilino et al. 2011	Gar3
QC-16	HQ434734		FJ591109	*D. pulex*	NAPX	55.28	-77.75	Kuujjuarapik, Quebec	Canada	Vergilino et al. 2011	K52
QC-17	HQ434736, HQ434737		FJ591096	Hybrid *D. pulicaria* X *D. pulex*	NAPX	55.28	-77.75	Kuujjuarapik, Quebec	Canada	Vergilino et al. 2011	K92
QC-18	HQ434741, HQ434742, HQ434743		FJ591110	Hybrid *D. pulicaria* X *D. pulex*	NAPC	55.28	-77.75	Kuujjuarapik, Quebec	Canada	Vergilino et al. 2011	K228
QC-19	HQ434744		FJ591112	*D. pulicaria*	NAPC	55.28	-77.75	Kuujjuarapik, Quebec	Canada	Vergilino et al. 2011	K230
QC-20	HQ434745, HQ434747		FJ591111	Hybrid *D. pulicaria* X *D. pulex*	NAPC	55.28	-77.75	Kuujjuarapik, Quebec	Canada	Vergilino et al. 2011	K232
QC-21	HQ434749		HQ434663	Hybrid *D. pulicaria* X *D. pulex*	NAPX	48.37	-67.23	Metis, Quebec	Canada	Vergilino et al. 2011	Metis3
QC-22	EU918433, EU918434			*D. pulex*	NAPX	52.50	-74.00	Quebec	Canada	Omilian et al. 2008	FAT1_QUE
QC-23	EU918435, EU918436			*D. pulex*	NAPX	52.50	-74.00	Quebec	Canada	Omilian et al. 2008	GUY1_QUE
QC-24	EU918451, EU918452			*D.pulex*	NAPX	52.50	-74.00	Quebec	Canada	Omilian et al. 2008	STX1_QUE
QC-25		JN117741, JN117740		*D. pulicaria*	missing	55.28	-77.75	Kuujjuarapik, Quebec	Canada	Crease et al. 2011	C14_QC
QC-26		JN117735, JN117752		Hybrid *D. pulicaria* X *D. pulex*	NAPC	58.28	-77.75	Quebec	Canada	Crease et al. 2011	CX02_QC
QC-27		JN117784, JN117785		*D.pulex*	NAPX	47.44	-72.78	Quebec	Canada	Crease et al. 2011	X14_QC
QC-28		JN117786, JN117761		*D.pulex*	NAPX	46.37	-71.19	Quebec	Canada	Crease et al. 2011	X15_QC
QC-29		JN117761		*D.pulex*	NAPX	48.13	-69.17	Quebec	Canada	Crease et al. 2011	X22_QC
QC-30		JN117761, JN117790		*D.pulex*	NAPX	46.03	-73.45	Quebec	Canada	Crease et al. 2011	X23_QC
RUS-01	KC536343, KC536344		KC536563	*D. pulicaria*	EPC	68.06	53.58	Petchora Delta	Russia	this paper	DP083_004
RUS-02	KC536345, KC536346, KC536347		KC536564	*D. tenebrosa*	TEN	68.06	53.58	Petchora Delta	Russia	this paper	DP083_011
RUS-03			KC536564	*D. tenebrosa*	TEN	68.06	53.58	Petchora Delta	Russia	this paper	DP083_012
RUS-04			KC536564	*D. tenebrosa*	TEN	68.06	53.58	Petchora Delta	Russia	this paper	DP083_013
RUS-05			KC536571	*D. tenebrosa*	TEN	68.77	53.15	Petchora Delta	Russia	this paper	DP103_001
RUS-06			KC536572	*D. tenebrosa*	TEN	68.77	53.15	Petchora Delta	Russia	this paper	DP103_002
RUS-07			KC536572	*D. tenebrosa*	TEN	68.77	53.15	Petchora Delta	Russia	this paper	DP103_003
RUS-08	KC536348, KC536349, KC536350		KC536573	*D. tenebrosa*	TEN	68.06	53.58	Petchora Delta	Russia	this paper	DP104_001
RUS-09			KC536572	*D. tenebrosa*	TEN	68.06	53.58	Petchora Delta	Russia	this paper	DP104_002
RUS-10			KC536564	*D. tenebrosa*	TEN	68.06	53.58	Petchora Delta	Russia	this paper	DP104_003
RUS-11	KC536351, KC536352, KC536353	KC536060, KC536061, KC536062	KC536592	*D. tenebrosa*	TEN	68.77	53.15	Petchora Delta	Russia	this paper	DP160_002
RUS-12	KC536354, KC536355, KC536356		KC536592	*D. tenebrosa*	TEN	68.77	53.15	Petchora Delta	Russia	this paper	DP162_001
RUS-13	KC536357, KC536358, KC536359	KC536063, KC536064	KC536592	*D. tenebrosa*	TEN	68.77	53.15	Petchora Delta	Russia	this paper	DP162_003
RUS-14	KC536360, KC536361, KC536362		KC536592	*D. tenebrosa*	TEN	68.77	53.15	Petchora Delta	Russia	this paper	DP162_004
RUS-15	KC536471, KC536472	KC536099, KC536100	KC536606	*D. tenebrosa*	TEN	71.98	102.55	Khatanga	Russia	this paper	DX019_001
RUS-16			KC536606	*D. tenebrosa*	TEN	71.98	102.55	Khatanga	Russia	this paper	DX019_002
RUS-17	KC536473, KC536474		KC536606	*D. tenebrosa*	TEN	71.98	102.55	Khatanga	Russia	this paper	DX019_003
RUS-18			KC536606	*D. tenebrosa*	TEN	71.98	102.55	Khatanga	Russia	this paper	DX019_004
RUS-19	KC536475, KC536476, KC536477	KC536101, KC536102	KC536607	*D. tenebrosa*	TEN	74.92	112.62	Tsvetkov cape, Taimyr	Russia	this paper	DX020_001
RUS-20			KC536607	*D. tenebrosa*	TEN	74.92	112.62	Tsvetkov cape, Taimyr	Russia	this paper	DX020_002

RUS-21			KC536607	*D. tenebrosa*	TEN	74.92	112.62	Tsvetkov cape, Taimyr	Russia	this paper	DX020_003
RUS-22	KC536478, KC536479, KC536480	KC536103, KC536104	KC536608	*D. tenebrosa*	TEN	74.92	112.62	Tsvetkov cape, Taimyr	Russia	this paper	DX020_004
SJM-01	KC536389, KC536390, KC536391	KC536021, KC536022, KC536023	KC536579	*D. pulicaria*	NAPC	78.92	11.88	Storvatnet, Ny-Alesund	Svalbard	this paper	DP127_001
SJM-02	KC536392, KC536393, KC536394	KC536024, KC536025, KC536026	KC536580	*D. tenebrosa*	TEN	78.92	11.88	Storvatnet, Ny-Alesund	Svalbard	this paper	DP127_002
SJM-03	KC536395, KC536396, KC536397	KC536027, KC536028, KC536029	KC536581	*D. pulicaria*	NAPC	78.93	11.88	Solvatnet, Ny-Alesund	Svalbard	this paper	DP128_001
SJM-04			KC536581	*D. pulicaria*	NAPC	78.93	11.88	Solvatnet, Ny-Alesund	Svalbard	this paper	DP128_002
SJM-05	KC536398, KC536399, KC536400, KC536401	KC536030, KC536031	KC536582	*D. tenebrosa*	TEN	79.89	16.40	Mosselbukta	Svalbard	this paper	DP129_001
SJM-06			KC536582	*D. tenebrosa*	TEN	79.89	16.40	Mosselbukta	Svalbard	this paper	DP129_002
SJM-07	KC536402, KC536403, KC536404	KC536032, KC536033, KC536034	KC536583	*D. pulicaria*	EPC	78.11	15.90	Colesdalen	Svalbard	this paper	DP130_001
SJM-08			KC536583	*D. pulicaria*	EPC	78.11	15.90	Colesdalen	Svalbard	this paper	DP130_002
SJM-09	KC536405, KC536406, KC536407	KC536035, KC536036, KC536037	KC536584	*D. pulicaria*	NAPC	78.11	15.90	Colesdalen	Svalbard	this paper	DP130_003
SJM-10			KC536584	*D. pulicaria*	NAPC	78.11	15.90	Colesdalen	Svalbard	this paper	DP130_004
SK-01		JN117736, JN117737		*D. pulicaria*	NAPC	52.13	-105.13	Saskatchewan	Canada	Crease et al. 2011	C05_SK
SK-02		JN117740		*D. pulicaria*	NAPC	49.42	-105.14	Saskatchewan	Canada	Crease et al. 2011	C13_SK
SK-03		JN117742, JN117740		*D. pulicaria*	NAPC	49.42	-105.14	Saskatchewan	Canada	Crease et al. 2011	C14_SK
SK-04		JN117740		*D. pulicaria*	NAPX	50.92	-102.40	Saskatchewan	Canada	Crease et al. 2011	C17_SK
SK-05		JN117735, JN117767		Hybrid *D. pulicaria* X *D. pulex*	NAPX	50.07	-105.53	Saskatchewan	Canada	Crease et al. 2011	CX17_SK
SK-06		JN117735, JN117767		Hybrid *D. pulicaria* X *D. pulex*	NAPX	51.27	-103.72	Saskatchewan	Canada	Crease et al. 2011	CX18_SK
SK-07		JN117796, JN117797		*D.pulex*	NAPX	50.07	-105.53	Saskatchewan	Canada	Crease et al. 2011	X32_SK
SK-08		JN117767, JN117798		*D.pulex*	NAPX	49.85	-105.04	Saskatchewan	Canada	Crease et al. 2011	X33_SK
SK-09		JN117767, JN117799		*D.pulex*	NAPX	49.85	-105.04	Saskatchewan	Canada	Crease et al. 2011	X34_SK
SVK-01	KC536143, KC536144	KC535963, KC535964	KC536503	*D. pulicaria*	EPC	49.16	20.01	High Tatra Mts.	Slovak Republic	this paper	DP001_001
SVK-02	KC536145, KC536146			*D. pulicaria*	missing	49.16	20.01	High Tatra Mts.	Slovak Republic	this paper	DP001_003
SVK-03			KC536503	*D. pulicaria*	EPC	49.16	20.01	High Tatra Mts.	Slovak Republic	this paper	DP001_004
SVK-04			KC536503	*D. pulicaria*	EPC	49.16	20.01	High Tatra Mts.	Slovak Republic	this paper	DP001_006
SVK-05			KC536503	*D. pulicaria*	EPC	49.16	20.01	High Tatra Mts.	Slovak Republic	this paper	DP001_007
SVK-06	KC536147, KC536148		KC536503	*D. pulicaria*	EPC	49.16	20.01	High Tatra Mts.	Slovak Republic	this paper	DP001_008
SVK-07	KC536149, KC536150		KC536503	*D. pulicaria*	EPC	49.16	20.01	High Tatra Mts.	Slovak Republic	this paper	DP001_009
SVK-08	KC536151, KC536152		KC536504	*D. pulicaria*	EPC	49.16	20.01	High Tatra Mts.	Slovak Republic	this paper	DP001_010
SVK-09	KC536153, KC536154		KC536503	*D. pulicaria*	EPC	49.16	20.01	High Tatra Mts.	Slovak Republic	this paper	DP001_011
SVK-10			KC536503	*D. pulicaria*	EPC	49.16	20.01	High Tatra Mts.	Slovak Republic	this paper	DP001_012
SVK-11			KC536503	*D. pulicaria*	EPC	49.16	20.01	High Tatra Mts.	Slovak Republic	this paper	DP107_001
SVK-12			KC536503	*D. pulicaria*	EPC	49.16	20.01	High Tatra Mts.	Slovak Republic	this paper	DP107_006
SVK-13			KC536507	*D. pulicaria*	EPC	49.19	20.04	High Tatra Mts.	Slovak Republic	this paper	DP005_02M
SVK-14	KC536161, KC536162		KC536508	*D. pulicaria*	EPC	49.19	20.04	High Tatra Mts.	Slovak Republic	this paper	DP005_003
SVK-15			KC536508	*D. pulicaria*	EPC	49.19	20.04	High Tatra Mts.	Slovak Republic	this paper	DP005_004
SVK-16			KC536508	*D. pulicaria*	EPC	49.19	20.04	High Tatra Mts.	Slovak Republic	this paper	DP005_005
SVK-17			KC536507	*D. pulicaria*	EPC	49.19	20.04	High Tatra Mts.	Slovak Republic	this paper	DP005_006
SVK-18			KC536507	*D. pulicaria*	EPC	49.19	20.04	High Tatra Mts.	Slovak Republic	this paper	DP005_007
SVK-19	KC536163, KC536164		KC536508	*D. pulicaria*	EPC	49.19	20.04	High Tatra Mts.	Slovak Republic	this paper	DP006_09M
SVK-20			KC536508	*D. pulicaria*	EPC	49.19	20.04	High Tatra Mts.	Slovak Republic	this paper	DP109_001
SVK-21			KC536508	*D. pulicaria*	EPC	49.19	20.04	High Tatra Mts.	Slovak Republic	this paper	DP109_006
SVK-22			KC536508	*D. pulicaria*	EPC	49.19	20.04	High Tatra Mts.	Slovak Republic	this paper	DP110_001
SVK-23	KC536165, KC536166		KC536509	*D. pulicaria*	EPC	49.19	20.03	High Tatra Mts.	Slovak Republic	this paper	DP007_002
SVK-24			KC536506	*D. pulicaria*	EPC	49.19	20.09	High Tatra Mts.	Slovak Republic	this paper	DP009_003
SVK-25	KC536167, KC536168		KC536510	*D. pulicaria*	EPC	49.21	20.14	High Tatra Mts.	Slovak Republic	this paper	DP014_03M
SVK-26	KC536169, KC536170		KC536511	*D. pulicaria*	EPC	49.18	20.13	High Tatra Mts.	Slovak Republic	this paper	DP015_001
SVN-01	KC536267, KC536268		KC536535	*D. pulicaria*	EPC	46.41	13.81	Julian Alps	Slovenia	this paper	DP039_001
SVN-02	KC536269, KC536270	KC535989, KC535990, KC535991	KC536535	*D. pulicaria*	EPC	46.41	13.81	Julian Alps	Slovenia	this paper	DP039_005
SVN-03	KC536271, KC536272		KC536535	*D. pulicaria*	EPC	46.41	13.81	Julian Alps	Slovenia	this paper	DP039_006
SVN-04	KC536273, KC536274		KC536535	*D. pulicaria*	EPC	46.41	13.81	Julian Alps	Slovenia	this paper	DP039_007
SVN-05	KC536275, KC536276		KC536535	*D. pulicaria*	EPC	46.41	13.81	Julian Alps	Slovenia	this paper	DP039_008
SVN-06			KC536535	*D. pulicaria*	EPC	46.41	13.81	Julian Alps	Slovenia	this paper	DP039_009
SWE-01	KC536329, KC536330		KC536554	*D. pulex*	EPX	58.48	11.66	Munkedal	Sweden	this paper	DP076_002
SWE-02			KC536555	*D. pulex*	EPX	58.48	11.66	Munkedal	Sweden	this paper	DP076_003

SWE-03			KC536554	*D. pulex*	EPX	58.48	11.66	Munkedal	Sweden	this paper	DP076_004
SWE-04	KC536331, KC536332	KC536002, KC536003	KC536556	*D. pulex*	EPX	58.77	11.87	Furved	Sweden	this paper	DP077_003
SWE-05	KC536333, KC536334	KC536004, KC536005	KC536557	*D. pulex*	EPX	58.77	11.87	Furved	Sweden	this paper	DP077_004
SWE-06			KC536557	*D. pulex*	EPX	58.77	11.87	Furved	Sweden	this paper	DP077_005
SWE-07			KC536557	*D. pulex*	EPX	58.48	11.84	Kvarnhultsbacken	Sweden	this paper	DP078_002
SWE-08	KC536335, KC536336	KC536006, KC536007	KC536555	*D. pulex*	EPX	58.54	11.97	Rubbestad	Sweden	this paper	DP079_001
SWE-09			KC536555	*D. pulex*	EPX	58.54	11.97	Rubbestad	Sweden	this paper	DP079_002
SWE-10			KC536555	*D. pulex*	EPX	58.54	11.97	Rubbestad	Sweden	this paper	DP079_003
SWE-11	KC536337, KC536338		KC536558	*D. pulex*	EPX	58.81	11.86	Solberg	Sweden	this paper	DP080_001
SWE-12			KC536559	*D. pulex*	EPX	58.81	11.86	Solberg	Sweden	this paper	DP080_002
SWE-13			KC536559	*D. pulex*	EPX	58.81	11.86	Solberg	Sweden	this paper	DP080_003
SWE-14	KC536421, KC536422	KC536050, KC536051	KC536588	*D. pulex*	EPX	58.64	17.04	Oxelosund	Sweden	this paper	DP136_001
SWE-15			KC536588	*D. pulex*	EPX	58.64	17.40	Oxelosund	Sweden	this paper	DP136_002
SWE-16			KC536588	*D. pulex*	EPX	58.64	17.40	Oxelosund	Sweden	this paper	DP136_003
SWE-17	KC536423, KC536424	KC536052, KC536053	KC536589	*D. pulex*	EPX	62.88	18.45	Bonhamn	Sweden	this paper	DP151_001
SWE-18			KC536589	*D. pulex*	EPX	62.88	18.45	Bonhamn	Sweden	this paper	DP151_002
SWE-19			KC536589	*D. pulex*	EPX	62.88	18.45	Bonhamn	Sweden	this paper	DP151_003
SWE-20			KC536589	*D. pulex*	EPX	62.88	18.45	Bonhamn	Sweden	this paper	DP151_004
SWE-21	KC536425, KC536426	KC536054, KC536055	KC536590	*D. pulex*	EPX	55.71	13.21	Lund	Sweden	this paper	DP152_001
SWE-22			KC536590	*D. pulex*	EPX	55.71	13.21	Lund	Sweden	this paper	DP152_002
SWE-23			KC536590	*D. pulex*	EPX	55.71	13.21	Lund	Sweden	this paper	DP152_003
SWE-24	KC536427, KC536428	KC536056, KC536057	KC536590	*D. pulex*	EPX	55.72	13.44	Svarta	Sweden	this paper	DP153_001
SWE-25			KC536590	*D. pulex*	EPX	55.72	13.44	Svarta	Sweden	this paper	DP153_002
SWE-26			KC536590	*D. pulex*	EPX	55.72	13.44	Svarta	Sweden	this paper	DP153_003
WA-01	EU918539, EU918540			*D.pulicaria*	NAPC	47.61	-122.24	Washington	USA	Omilian et al. 2008	WASH_WA
WA-02		JN117735		*D.pulicaria*	NAPX	47.61	-122.24	Washington	USA	Crease et al. 2011	C11_WA
WI-01	EU918449, EU918450			*D.pulex*	NAPX	44.50	-89.50	Wisconsin	USA	Omilian et al. 2008	SHK103_WI
WI-02	EU918543, EU918544			*D.pulicaria*	NAPC	44.50	-89.50	Wisconsin	USA	Omilian et al. 2008	WIND2_WI
WI-03		JN117771, JN117761		*D.pulex*	NAPX	46.67	-90.92	Wisconsin	USA	Crease et al. 2011	X03_WI
WI-04		JN117778, JN117788		*D.pulex*	NAPX	45.66	-88.10	Wisconsin	USA	Crease et al. 2011	X20_WI
WY-01		JN117738, JN117739		*D. pulicaria*	NAPC	44.55	-110.38	Yellowstone Lake, Wyoming	USA	Crease et al. 2011	C06_WY
XX-01	HQ434692, HQ434693			*D. pulicaria*	NAPC	missing	missing	missing	missing	Vergilino et al. 2011	A24_204
